# Context Matters: Patterns in Physical Distancing Behavior Across Situations and Over Time During the Covid-19 Pandemic in the Netherlands

**DOI:** 10.1093/abm/kaad053

**Published:** 2023-09-14

**Authors:** Carlijn Bussemakers, Mart van Dijk, Bas van den Putte, Marijn de Bruin

**Affiliations:** Institute of Health Sciences, IQ Healthcare, Radboud University Medical Center, Nijmegen, The Netherlands; Centre for Prevention, Lifestyle and Health, National Institute for Public Health and the Environment, Bilthoven, The Netherlands; Amsterdam School of Communication Research (ASCoR), University of Amsterdam, Amsterdam, The Netherlands; Institute of Health Sciences, IQ Healthcare, Radboud University Medical Center, Nijmegen, The Netherlands; Centre for Prevention, Lifestyle and Health, National Institute for Public Health and the Environment, Bilthoven, The Netherlands

**Keywords:** Physical distancing, Environmental opportunities and restrictions, Social motivations, Sociodemographic differences, Covid-19 pandemic, Dynamic cohort study

## Abstract

**Background:**

Physical distancing is an effective preventative measure during respiratory infectious disease outbreaks. Prior studies on distancing behaviors have largely ignored context characteristics (physical, social) and time.

**Purpose:**

We investigated patterns in physical distancing over time and across situations, as well as sociodemographic variation herein.

**Methods:**

We employed data from five rounds of a cohort study conducted throughout the pandemic by the Dutch public health institute (RIVM; *N* ≈ 50.000 per round). We conducted Latent Class Analyses to investigate patterns of physical distancing in a range of situations, followed by regression models to investigate associations between distancing behavior and sociodemographic and context characteristics.

**Results:**

Participants differed in their general tendency to adhere to distancing guidelines across situations, but there were also substantial differences in distancing behavior between situations, particularly at work, with friends and family and outdoors. Distancing at work was strongly associated with work environment characteristics. Younger age groups reported less distancing behavior, particularly with close relations (friends or family) and at work. In periods when the pandemic situation was most severe, people adhered more strongly to distancing guidelines and age differences were most pronounced during these periods.

**Conclusions:**

Physical and social context matters for physical distancing, highlighting the importance of developing strategies for pandemic preparedness that improve opportunities for physical distancing (e.g., reducing crowding, one-way traffic) and accommodate young people to safely meet even in times of high pandemic severity and lockdowns. Future studies should account for the physical and social context in which distancing behavior is observed.

## Teaser

Participants differed in their general tendency to adhere to distancing guidelines across situations, but there were also substantial differences in distancing behavior between situations, particularly at work, with friends and family and outdoors. Distancing at work was strongly associated with work environment characteristics, while younger age groups reported less distancing behavior, particularly in social situations.

## Introduction

To reduce the spread of Covid-19, the Dutch government like many other governments advised or required people to keep a safe distance from people who were not part of their own household, in the Dutch case 1.5 m. Physical distancing measures can reduce virus transmission, but its effectiveness depends strongly on public adherence to these guidelines [1, 2]. The literature on physical distancing behavior has thus far mainly studied between-person variation in general physical distancing behavior (i.e., staying home or distancing from others across all situations) or distancing in one specific situation such as the workplace or the supermarket [3–5]. Although it is very likely that some people adhere more strongly to physical distancing guidelines than others across situations, this approach can obscure important differences in people’s distancing behavior caused by differences in the social and physical context. In other words, regardless of people’s general willingness to keep their distance, their capabilities, opportunities, and motivation to do so may differ from situation to situation [6, 7]. Previous work, for instance, has shown that people were less willing to distance from family and friends than from strangers [8]. Similarly, contexts differ in the physical opportunities they provide to distance, for instance because some are more crowded than others [9, 10].

An unanswered question is how such differences between situations translated into differences in people’s physical distancing behavior between situations. We expected that in addition to general between-person differences in physical distancing behavior across situations [3, 11], there would also be population subgroups who differed in their physical distancing behavior in specific situations. Some people may for instance have been more capable or motivated to distance in situations that offered less opportunities to do so [6], for instance because they had the possibility to shop during quiet hours or because they felt more comfortable asking others to keep their distance. Conversely, for some people, the need for close social contact with friends or family may have surpassed their motivation to adhere to physical distancing guidelines, while others were less affected by this. We therefore also expected that people with similar overall tendencies to distance, differed in their behavior in specific situations (e.g., some people kept their distance from friends and family in social situations whereas others were more likely to do so from colleagues at work). The Corona Behavioral Unit Cohort Study in the Netherlands provided the unique opportunity to unravel such patterns because it asked participants about their physical distancing behaviors across a range of situations and social relations (e.g., at work, when visiting friends or family, or in public spaces, as well as avoiding crowds which is a more general way to limit proximity to others) during different waves of the Covid-19 pandemic. With these data, we aim to answer the following research question: *how did people differ in their physical distancing behavior in different situations?* (RQ1).

Secondly, we explored whether people with different physical distancing behavior patterns differed with respect to sociodemographic characteristics. Multiple studies indicated that sociodemographic characteristics were associated with overall adherence to physical distancing guidelines, showing for instance that women, older people, and higher educated people were more likely to distance from others [6, 12]. However, this may not show the full picture, as sociodemographic differences may also vary between situations. Previous work indicates that differences in distancing behavior may partly be due to variation in the practical obstacles people face, for instance because people with a lower socioeconomic status more often live and work in situations where it is more difficult to physically distance from others [6]. This indicates that some sociodemographic groups may have been more strongly affected by (a lack of) opportunities to distance in specific situations. Similarly, social groups may have varied in how strongly social factors such as the need for social contact affected their distancing behavior. It is likely that groups with a higher need for social contact, such as people who lived alone and young people (for whom Covid-19 was also perceived as less dangerous) [13], were less motivated to physically distance in social situations with friends or close relatives [11, 14]. To gain a better understanding of sociodemographic differences in distancing behavior, we also ask: *how were sociodemographic characteristics associated with physical distancing behavior in different situations?* (RQ2).

Our third research question focused on whether during the course of the Covid-19 pandemic, physical distancing behavior in different situations (RQ3a) and sociodemographic variation herein (RQ3b) changed. Two factors likely influenced distancing behavior over time: the severity of the Covid-19 situation and time passed since the start of the pandemic [15, 16]. Regarding the first, we expected that more people adhered to physical distancing measures when the pandemic situation was more severe (i.e., more Covid-19 cases), when measures were typically also more stringent (including those focused on physical distancing). Conversely, we expected that fewer people adhered to distancing guidelines as the pandemic progressed, because of an increased need for social contact, higher levels of immunity (due to vaccinations and prior infections), and possibly pandemic fatigue [4, 15]. Sociodemographic differences in physical distancing behavior could also have become more pronounced over time and in periods with lower pandemic severity [12]. In such situations, the relative need to distance became smaller, while the need for contact increased and social norms around physical distancing relaxed. We expected this would make the above sociodemographic differences more pronounced.

Because the results of our analyses of patterns in physical distancing (RQ1 and RQ3a) affected the analytical approach to studying sociodemographic variation (RQ2 and RQ3b), we discuss methods and results of these two parts of the study separately. First, we discuss the methods and results of studying patterns of physical distancing across situations and rounds, followed by the methods and results of studying sociodemographic variation in distancing behavior and variation herein over time. A preregistration of the study was published at OSF (https://osf.io/wq7hn/). Study method and results are reported following the Strengthening the Reporting of Observational Studies in Epidemiology (STROBE) Statement.

## Patterns of Physical Distancing Across Situations and Rounds: Materials and Method

### Data

We used data from the Corona Behavior & Well-being cohort study, a dynamic cohort study conducted in 21 rounds between April 2020 and September 2022 among the Dutch population by the Behavioral Unit of the National Institute for Public Health and The Environment (RIVM) [17]. We selected five rounds of the survey that strongly differed with respect to the severity of the Covid-19 situation as well as the vaccination rate, to represent the different stages of the pandemic in the Netherlands. Table 1 provides information on the period in which these five rounds were conducted.

**Table 1:** Information on the Study Rounds Included in the Analysis

Round	Date	Severity (number of infections and policy stringency)	Mass vaccination^a^
2	May 7–12, 2020	High (1,100–1,189 people hospitalized, policy stringency indexed at 78.7)	Nobody vaccinated
5	July 8–12, 2020	Low (79–83 people hospitalized, policy stringency indexed at 45.37)	Nobody vaccinated
11	March 24–28, 2021	High (1,562–1,593 people hospitalized, policy stringency indexed at 75)	Some people vaccinated (8% of total population received at least one dose)
16	October 20–24, 2021	Low (485–563 people hospitalized, policy stringency indexed at 35.72)	Most people vaccinated (70% of total population received at least one dose, 65% completed the initial protocol)
18	January 19–23, 2022	High (818–846 people hospitalized, policy stringency indexed at 50.32)	Most people vaccinated (72% of total population received at least one dose, 67% completed the initial protocol, 47% also received a booster dose)

^a^Hospitalizations retrieved from the Dutch National Government [18], Policy stringency (Oxford stringency index) and vaccination data retrieved from Our World in Data [19].

### Analytical Strategy: Latent Class Analysis

To study patterns of physical distancing behavior across situations, we conducted Latent Class Analysis (LCA) using the poLCA (Polytomous Variable Latent Class Analysis) package in R [20]. We used two criteria to select the most appropriate number of classes in each round: model fit as indicated by BIC and AIC (lower values indicating better fit) [20] and substantive interpretability [21]. Additionally, entropy of the optimal latent class solution should be higher than 0.6, since lower values indicate poor separation of classes [21]. To examine variation over time, we conducted the LCA for each of the five rounds separately.

### Measures Included in the LCA

**Fig. 1.**
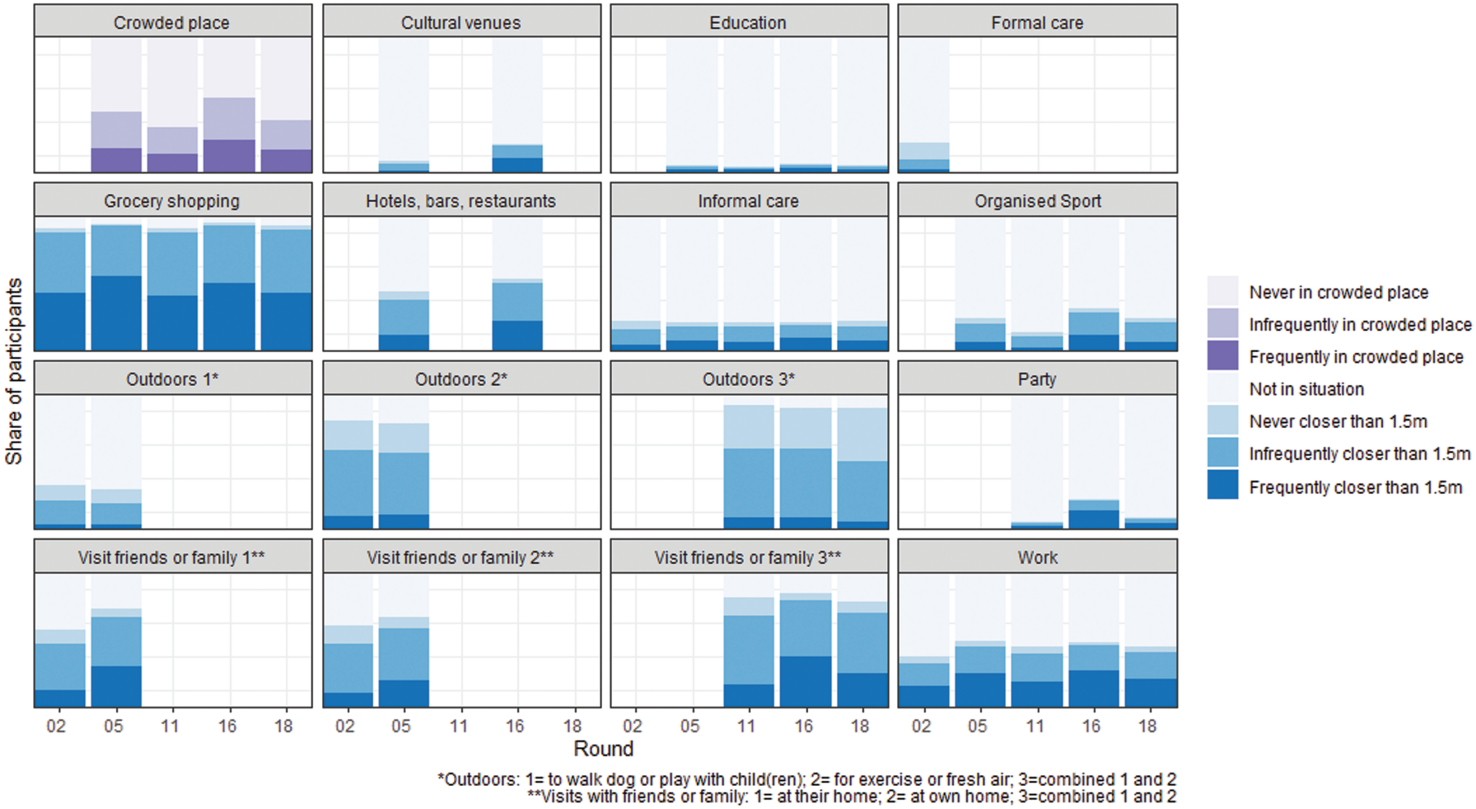
Physical distancing behavior across rounds.

To illustrate patterns of physical distancing, we included measures of physical distancing in different situations as well as avoiding crowds in the LCA. The measures of physical distancing in specific situations were each based on two questions. The first question asked participants to indicate how often they left the house to go to a certain setting or for a specific activity in the past 7 days. A follow-up question for people who indicated they did so at least once asked them how often others came closer than 1.5 m the last time they left the house to go to this setting or did this activity (never, seldom, sometimes, regularly, often, or very often). See Fig. 1 and Appendix B for an overview of which situations were included in each round. For each situation, we combined participants’ answers to these two questions into one variable with four categories:

1) “Has not been in situation” (participants who did not leave the house for a certain setting or specific activity in the past week)
2) “Never within 1.5 m distance from others” (participants who had been in the situation at least once in the past week and indicated others “never” came closer than 1.5 m)
3) “Infrequently closer than 1.5 m from others” (participants who had been in the situation at least once in the past week and indicated others “seldom” or “sometimes” came closer than 1.5 m)
4) “Frequently closer than 1.5 m from others” (participants who had been in the situation at least once in the past week and indicated others ‘regularly or “(very) often” came closer than 1.5 m).

The “not in situation” category included people who decided to avoid a situation for physical distancing reasons as well as people who would not be in that situation regardless of physical distancing guidelines, so we cannot draw strong conclusions about this group. It is, however, important to include the “not in situation” category in the LCA because excluding these participants would result in a very small, highly selective sample of participants who had been in all situations included in the questionnaire.

Avoiding crowded places was measured by asking participants how often they had gone to a place that turned out to be too crowded to keep 1.5 m distance in the past week. If people responded yes, a follow-up question asked how often they left or went elsewhere because the place was too crowded. We subtracted the number of times participants left such a place from the total number of times they had been in this situation. Because the variable is highly skewed, we categorized answers in three categories: never, infrequently (below average frequency) and frequently (above average frequency).

### Missing Values and Sample Description

Participants who did not answer any question on physical distancing were excluded from the LCA (7.14% of participants in Round 2, none in the other rounds), participants with missing data on some of these questions remained in the analyses. Table 2 provides the sociodemographic composition of the sample. Because the composition is highly similar across rounds, we present the information of all rounds combined. Appendix A provides the sample composition per round.

**Table 2:** Sociodemographic Sample Characteristics (Rounds Combined)

		*N*	%
Sex	Male	107,049	38.11
	Female	173,790	61.88
	Other	27	0.01
Age	70+	57,857	20.60
	55–69	100,436	35.76
	44–54	79,237	28.21
	25–39	39,193	13.95
	16–24	4,143	1.48
Education level	Low (including no education)	33,345	11.87
	Middle	75,373	26.84
	High	170,143	60.58
	Unknown	2,005	0.71
Born in	Netherlands	267,391	95.20
	Elsewhere	12,981	4.62
	Unknown	494	0.18
Lives with: partner	No	79,388	28.27
	Yes	201,478	71.73
Lives with: child(ren)	No	206,536	73.54
	Yes	74,330	26.46
Lives with: other adult(s)	No	239,564	85.29
	Yes	41,302	14.71
Underlying medical conditions	No	211,086	75.16
	Yes	69,658	24.80
	Unknown	122	0.04
Employment	Not employed	99,558	35.45
	Employed, not essential worker/vital sector	107,411	38.24
	Employed, essential worker/vital sector	73,897	26.31
In education	No	272,765	97.12
	Yes	8,101	2.88
Urbanization municipality	<500	17,008	6.06
	500–1,000	50,297	17.91
	1,000–1,500	41,680	14.84
	1,500–2,500	84,444	30.07
	2,500	87,362	31.10
	Unknown	75	0.03
Socioeconomic status municipality	Very low	29,970	10.67
	Low (including no education)	67,185	23.92
	Intermediate-low	38,685	13.77
	Intermediate-high	62,128	22.12
	High	59,659	21.24
	Very high	22,967	8.18
	Unknown	272	0.10

## Patterns of Physical Distancing Across Situations and Rounds: Results

### Description of Physical Distancing Behavior Across Situations and Rounds

Figure 1 provides descriptive statistics of physical distancing behavior in different situations across rounds (Appendix B provides the same information in a table). There were substantial differences in people’s distancing behavior between situations. Participants were most likely to have been within 1.5 m from others when grocery shopping: almost all participants had been grocery shopping and, in all rounds, more than 40% reported frequently being within 1.5 m from others when doing so. Frequently being within 1.5 m from others was also relatively common among participants who went to work (>40% in all rounds) and parties (e.g., >60% in Round 16). However, it has to be noted that most participants did not go to a party, which could be because they did not have a party last week or because they actively avoided parties which can be a form of distancing behavior. Distancing was most common outdoors, as less than 15% of participants who went outdoors indicated that they frequently came close to others.

We also found several relevant changes over time. During periods with fewer infections and less stringent policies, participants avoided crowded places less often (45% in Round 16 vs. more than 60% in 11 and 18). In such periods, participants also attended social situations more often (e.g., 22% of participants attended a party in Round 16, compared with <10% in Rounds 11 and 18) and were more frequently within 1.5 m distance from others in such situations (e.g., 60% at parties in Round 16 compared with 32% in Round 11; 45% when visiting with friends or family in Round 16 compared with 20% in Round 11).

### LCA of Physical Distancing Behavior Across Situations and Rounds

**Fig. 2.**
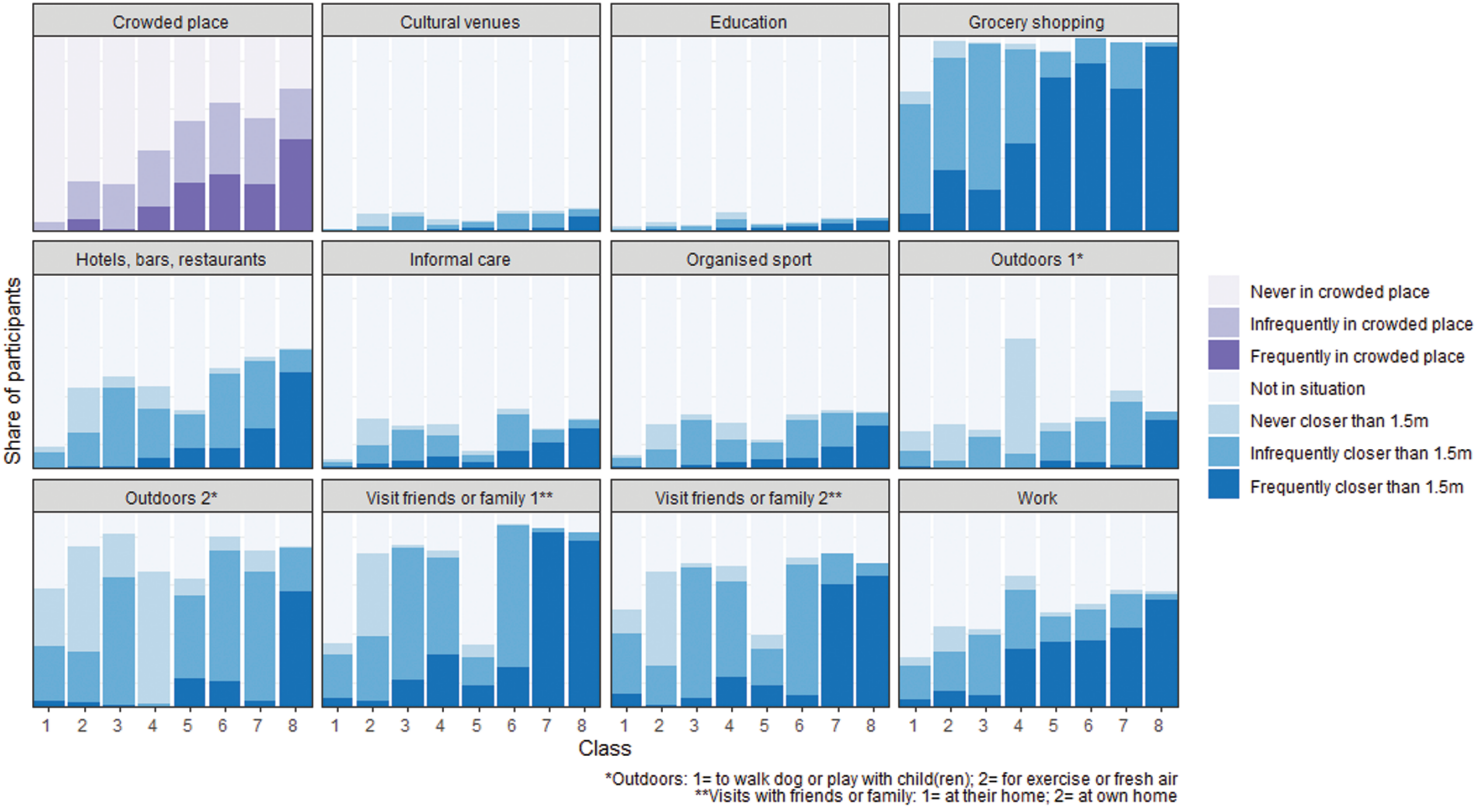
Physical distancing behavior of eight latent classes (Round 5).

We found that a model with eight classes was most appropriate for Round 5, because it had a good model fit (relatively low BIC and AIC) and substantive interpretation, as well as sufficient entropy (0.61). Although solutions with more classes had slightly better model fit as indicated by lower BIC and AIC values, the improvement in model fit was marginal, while these models had lower entropy and did not reveal relevant distinctions that were not present in the solution with eight classes. Figure 2 presents the results of the model with eight classes, a detailed discussion of the LCAs and their interpretation for all rounds can be found in Appendix C. Overall, this model distinguished classes based on general distancing behavior (across situations), with additional distinctions due to distancing behavior in specific situations.

The increasing darker bars from Class 1 to 8 in Fig. 2 indicate that classes mainly differed in general distancing behavior: some classes consisted mostly of participants who were seldom within 1.5 m from others (Classes 1 and 2), while Class 8 included participants who were in crowded places most often and most often reported being within 1.5 m distance from others across situations. The other classes were in-between these two extremes.

Within these classes with similar general distancing behavior, there were differences in behavior when visiting with friends or family, outdoors and at work, as well as their mobility. Of the classes who strongly followed distancing guidelines (Classes 1 and 2), Class 1 (10%) included a large share of people who mainly stayed at home and avoided crowded places (i.e., low mobility), while Class 2 had higher mobility, but was very likely to keep 1.5 m distance from others. Class 2 was even more likely to distance when outdoors and when visiting with friends or family than Class 1. Among the two classes with slightly lower levels of distancing (Classes 3 and 4), similar differences were found as among the classes who very strongly followed distancing guidelines: Class 3 (14%) included participants who had relatively low mobility (especially for avoiding crowded places, outdoors and at work) while Class 4 (15%) included participants who left their homes more often than Class 3 and were very likely to distance outdoors, but somewhat less so when visiting with friends or family and at work. Classes 5, 6, and 7 had moderate overall levels of distancing and mainly differed in distancing behavior in social situations, such as when visiting friends or family: Class 5 (15%) was less often in social situations than Class 6 (17%), while Class 7 (14%) was more often within 1.5 m distance from others in such situations.

Contrary to Round 5, for Rounds 2, 11, 16, and 18 it was unfortunately not possible to select an optimal latent class solution, as none of the models had both good model fit and sufficient entropy (see Appendix C). However, for each round, we found several latent class solutions with a relatively low BIC that showed similar distinctions as the 8-class model of Round 5. All models with relatively good model fit mainly distinguished classes based on general distancing behavior, with additional distinctions based on distancing when visiting with friends or family, or at work. In Round 11, classes were also distinguished based on how often they were within 1.5 m from others outdoors. So, although there was not one optimal latent class solution for these rounds, the results aligned with the optimal model from Round 5.

## Sociodemographic Variation in Physical Distancing: Materials and Method

Due to the relatively low entropy of our LCA models, it was not possible to use the latent classes as dependent variables to study sociodemographic variation in physical distancing behavior [21]. To gain insight in how distancing sociodemographic characteristics relate to difference in distancing behavior and how this changed over time, we therefore opted for an alternative approach by separately regressing four distancing variables on the sociodemographic characteristics.

### Measures Included in the Regression Models

The choice of the four distancing variables was based on the factors that most strongly influenced distinctions between latent classes: general distancing behavior as well as distancing in three specific situations: when visiting with friends or family, at work, and outdoors (net of general distancing behavior).

#### Dependent variables

Because distancing was more difficult in some situations than others (see Fig. 1), it was important to account for the specific situations participants had been in when measuring their general distancing behavior. We therefore first mean-centered the physical distancing variable of each situation (based on participants who had been in that situation). Second, we calculated the general distancing behavior of each participant by calculating the average of these mean-centered variables (with higher scores indicating more violations). In this way, the measure indicated whether participants were relatively less likely to distance than others while accounting for the specific situation(s) they had been in.

In the analyses of distancing in the three specific situations, we used the categorical, situation-specific variables of distancing with friends or family, outdoors, and at work (never, infrequently, and frequently within 1.5 m from others) as dependent variables. For distancing outdoors and with friends or family, we combined the two questions asked in Rounds 2 and 5 into one measure for each situation, to align with the questions included in the other rounds. Most participants (between 86% and 95% per situation and round) reported the same behavior for the two measures, participants who did not report the same behavior received their highest score of the two original variables on the combined measure.

#### Sociodemographic and control variables

As independent variables, we included all sociodemographic characteristics available in the questionnaire. These were the following categorical variables: sex (male and female), age (70+, 55–69, 44–54, 25–39, and 16–24), education level (low, middle, and high), country of origin (born in the Netherlands and born elsewhere), living situation (three separate dichotomous variables indicating participant lived with their partner, children under 18, and other adults), underlying medical conditions (no or yes), employment including self-employment and volunteer work (not employed, employed but not an essential worker or in a vital sector, and employed as an essential worker or in a vital sector), being in education (no or yes), urbanization of participants’ municipality (<500, 500–1,000, 1,000–1,500, 1,500–2,500, and >2,500 addresses per km²) and socioeconomic status of the municipality (six categories ranging from very low to very high). We also include a measure of (a lack of) opportunities for distancing in participants’ specific context: participants were asked whether they were regularly within 1.5 m due to their work in health care, providing informal care, and work activities or their physical work environment. These were included as three separate dichotomous variables in the analyses of general distancing and distancing at work.

Two sociodemographic characteristics were only available in specific rounds and were therefore included in additional analyses. Having an outdoor space at home (such as a garden) was coded as a dichotomous variable and included in the analyses of physical distancing with friends or family in participants’ own home in Rounds 2 and 5. Employment sector was included in Rounds 11 and 18, albeit with a more extensive list of sectors in Round 18, and included in the analysis of physical distancing at work for those two rounds. All original answer categories were included in the additional analyses.

Finally, we included four dichotomous control variables indicating situations that required participants to quarantine or isolate during the past 6 weeks (in line with Dutch policy at that time), as this may have overlapped with the past week concerned in the questions on physical distancing. These were having Covid symptoms (all rounds), testing positive for Covid (Rounds 11, 16, and 18), a household member who tested positive for Covid (Rounds 11 and 18) and having been in close contact with someone who tested positive (Rounds 11 and 18).

### Regression Models for Sociodemographic Differences in Physical Distancing Behavior

Because general distancing behavior was a continuous variable, we used OLS regression models for this outcome. Distancing in the three specific situations had three categories (“never,” “infrequently,” and “frequently” violating distancing guidelines) and was therefore analyzed with multinominal logit models. We included general distancing behavior in other situations as a control variable in these latter three models, to shed light on how sociodemographic characteristics are associated with distancing behavior specifically in these three situations (rather than being an expression of people’s general tendency to distance more or less). Because logistic regression coefficients are not comparable between samples and models, we present average marginal effects which can be interpreted as the difference in the probability of a specific outcome [22]. We consider effects larger than (−)0.1 to be substantial, as this value corresponds to an effect size of 10% in the average marginal effects and >0.2 standard deviation in the OLS models [23]. Regression models were conducted for each round separately to illustrate variation over time.

### Missing Values and Sample Size

For the regression models of general distancing behavior, participants who had not been in any situation (0.62% of the total sample) and participants with missing data on at least one sociodemographic variable were excluded (1.63% of the total sample). The three regression models for specific situations only included participants who had been in that situation. The sample size per outcome variable can be found in Tables 3 and 4.

**Table 3:** Descriptive Statistics of General Physical Distancing Behavior

	*N*	Mean	Std. dev.	Min	Max
Round 2	51,766	−0.04	0.44	−1.46	1.52
Round 5	45,869	0.05	0.42	−1.46	1.23
Round 11	45,959	−0.09	0.43	−1.46	1.23
Round 16	37,503	0.07	0.43	−1.46	1.23
Round 18	40,638	−0.07	0.44	−1.46	1.23

**Table 4:** Descriptive Statistics of Physical Distancing in Specific Situations

	Round 2		Round 5		Round 11		Round 16		Round 18	
	*N*	%	*N*	%	*N*	%	*N*	%	*N*	%
Visiting friends or family	40,202		39,813		37,455		24,957		25,590	
Never	7,206	13.92	3,208	6.99	6,272	13.65	1,509	4.02	2,615	6.43
Infrequently	24,122	46.60	20,308	44.27	23,669	51.50	12,243	32.65	14,764	36.33
Frequently	8,874	17.14	16,297	35.53	7,514	16.35	11,205	29.88	8,211	20.21
Work	19,282		22,230		20,566		18,134		18,202	
Never	2,460	4.75	1,761	3.84	2,123	4.62	897	2.39	1,528	3.76
Infrequently	8,669	16.75	8,883	19.37	9,899	21.54	6,840	18.24	8,310	20.45
Frequently	8,153	15.75	11,586	25.26	8,544	18.59	10,397	27.72	8,364	20.58
Outdoors	46,626		39,829		42,220		33,478		36,525	
Never	12,543	24.23	10,791	23.53	14,847	32.30	11,492	30.64	16,277	40.05
Infrequently	28,206	54.49	23,657	51.58	23,711	51.59	18,979	50.61	18,096	44.53
Frequently	5,877	11.35	5,381	11.73	3,662	7.97	3,007	8.02	2,152	5.30

## Sociodemographic Variation in Physical Distancing: Results

### Description off Physical Distancing Across Situations for Regression Models

Tables 3 and 4 present descriptive statistics of the physical distancing variables as used in the regression models. Note that the general distancing measure presented in Table 3 is based on participants’ average score on standardized situation-specific distancing variables (see dependent variables). It is therefore not possible to provide a meaningful interpretation of the mean values for each round, but higher scores indicate that participants were relatively more often within 1.5 m from others compared with the other rounds. Overall, the patterns confirm the changes over time shown in Fig. 1: in periods with lower pandemic severity, participants were more often within 1.5 m from others (Rounds 5 and 16 each have 0.14 higher average than the next round, a difference of 1/3 standard deviation). Furthermore, Table 4 shows that people were more often within 1.5 m distance from friends or family and at work in periods with lower severity (Rounds 5 and 16) as well as in a later stage of the pandemic (Round 18). Notably, distancing behavior outdoors seems to increase during the pandemic, as more people reported never violating distancing guidelines outdoors in later rounds, possibly because outdoor spaces were relatively popular and thus crowded during the early stages of the pandemic.

### Regression Models for Sociodemographic Variation in Physical Distancing

**Fig. 3.**
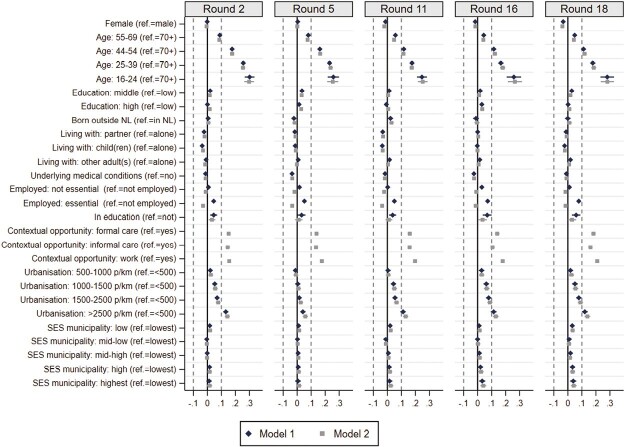
Associations between sociodemographic factors and typical physical distancing (higher scores indicate more frequently within 1.5 m from others).

Figure 3 presents the associations between sociodemographic characteristics and general physical distancing in the five rounds under study. Model 1 includes the sociodemographic indicators; in Model 2 opportunities to distance at work and when providing informal care were also included. The figure presents unstandardized effects because all independent variables were categorical. Although most effects were statistically significant, they tended to be (very) small. Only age, work environment (opportunities to distance at work), and urbanization had a substantial effect (>0.1). Substantial age differences were found in each round, with younger age groups reporting more often being within 1.5 m distance from others than older age groups. This pattern remained stable throughout all five rounds representing different phases of the pandemic. Similarly, the effects of urbanization indicated that people living in the most densely populated municipalities were more likely to be close to others, which was found in all rounds except Round 5, a period with low severity. Finally, the work environment and activities played an important role, as people who reported that their work or informal care obligations made that they often could not keep 1.5 m distance from others were generally more often within 1.5 m distance from others.

**Fig. 4.**
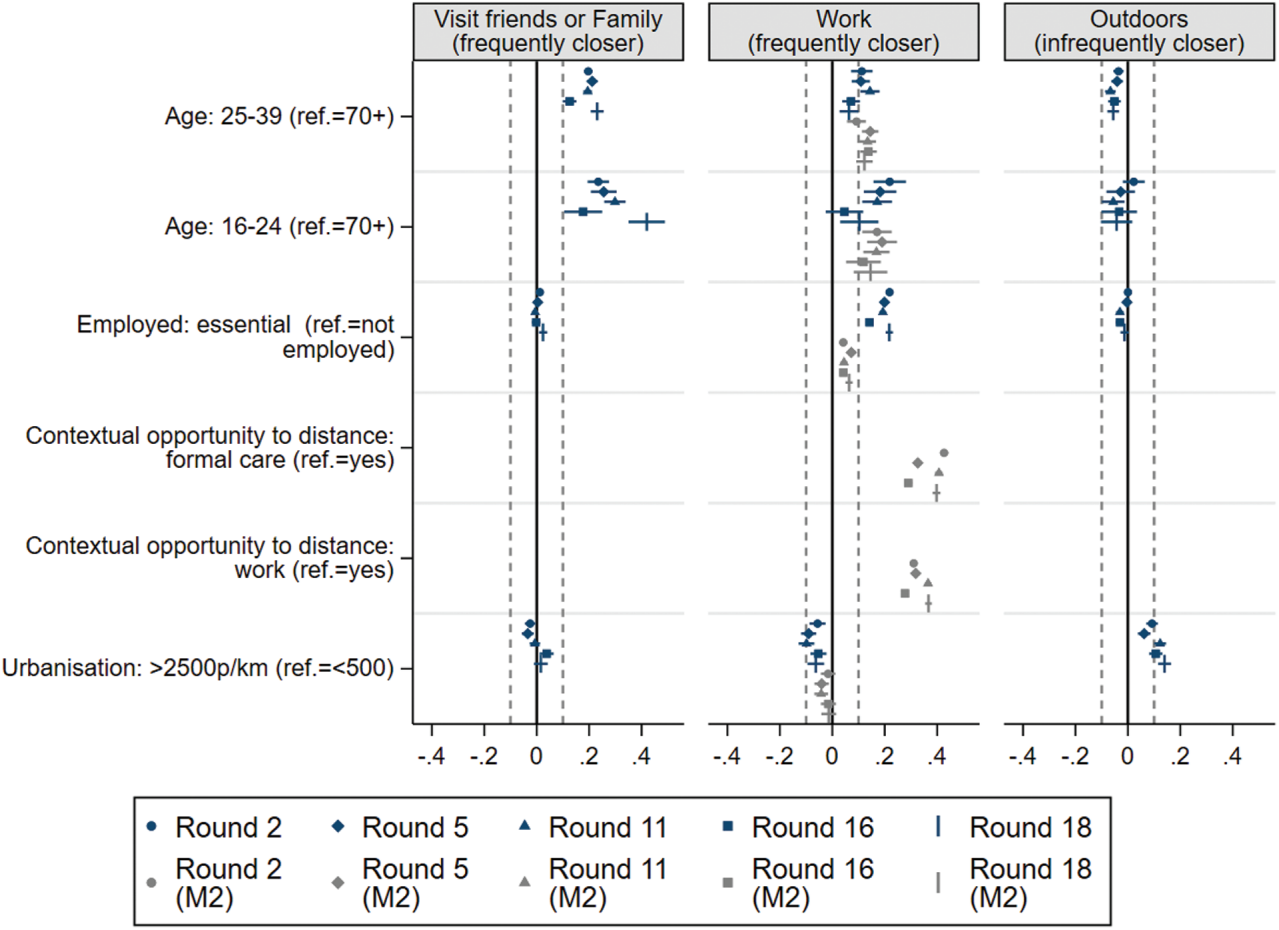
Substantial associations between sociodemographic factors and situation-specific physical distancing (average marginal effects). (Full models including all predictors can be found in Appendix D.)

We also estimated associations between sociodemographic characteristics and distancing behavior in specific situations (controlled for general distancing in other situations), of which the most important associations are presented in Fig. 4. Because the outcome variables were categorical, the figures present average marginal effects, which refer to the change in the probability of an outcome, relative to the other two outcomes. For distancing with friends or family and at work, we present the average marginal effects for frequently being within 1.5 m from others, as this is the riskiest form of nonadherence to distancing guidelines. For distancing outdoors, however, frequently being within 1.5 m from others was very uncommon (see Table 4), so we focus on infrequently violating guidelines. Average marginal effects for all outcome categories can be found in Appendix D.

Most sociodemographic variables had small effects, except for age, employment/work environment and urbanization, indicating that these factors were not only relevant for general distancing, but particularly for distancing in specific situations. Younger age groups (16–24 and 25–39 years old) were more likely to frequently violate distancing guidelines when visiting with friends or family and at work. The work context was only relevant for distancing at work, as people with essential employment were more likely to frequently be within 1.5 m from others at work, which was fully explained by a lack of opportunities to keep distance from others at work. For distancing outdoors, only urbanization had a clear impact with lower levels of distancing outdoors among people from the most densely populated municipalities. Of these associations, the impact of age when visiting with friends or family changed over time: age differences in distancing with friends or family were largest in Round 18 and smallest in Round 16, indicating that differences were most profound when the Covid situation was relatively severe.

The additional analyses can be found in Appendix E and show that having a private outdoor space such as a garden or balcony was not associated with distancing when friends or family came to visit. We found small differences in distancing at work between participants employed in different sectors (e.g., less distancing in health care or education), which were almost fully explained by a lack of opportunity to keep 1.5 m distance due to participants’ work or work environment.

## Discussion

### Main Findings

In this study, we investigated variation in people’s physical distancing behavior, as well as differences herein between sociodemographic groups and different phases of the pandemic. Our Latent Class Analyses indicated that people mainly differed in their general physical distancing behavior: across all situations, some classes were more likely to distance than others. Some contexts provided better opportunities for physical distancing than others, and these situational differences were largely similar across classes: all classes were for example relatively likely to distance outdoors and unlikely to do so when grocery shopping. We also found evidence that participants with similar overall levels of physical distancing differed in distancing behavior when visiting with friends or family, at work and outdoors. So, although participants mainly differed in general distancing behavior displayed across all situations, there were situations in which people deviated from these overall tendencies.

Our regression models with sociodemographic factors indicated that both differences in opportunities and motivations may play a role for these situation-specific distancing behaviors. Participants working in sectors such as health care and education had lower levels of distancing at work because their work activities and environments made it impossible to keep 1.5 m distance from others. Similarly, participants in more densely populated municipalities were more likely to be within 1.5 m distance from others outdoors, likely due to crowdedness. These findings align with previous work indicating that distancing practices such as avoiding contact by working from home differ markedly between socioeconomic groups, highlighting the importance of differential opportunities for physical distancing between people with different sociodemographic backgrounds [6]. Moreover, younger age groups were more often within 1.5 m distance from others, particularly with friends or family and at work. This aligns with findings from previous studies indicating young people had lower motivation for physical distancing because they faced a relatively lower risk of serious illness due to Covid-19 as well as an increased need for social contact [4, 15]. Moreover, the intention-behavior gap seemed to be larger for younger people, indicating that even with high motivation, they may not keep their distance from others [24]. Unlike previous studies, we did not find strong associations between other sociodemographic factors and physical distancing, such as between men and women, or between lower and higher educated people. The reason why we did not find such differences may be because we accounted for the different situations participants had been in. The overall tendency of higher educated people to physically distance from others may for instance be overestimated in previous studies because their jobs made it easier to work from home or keep their distance from others [6, 11], while they were not more likely to physically distance in other situations.

Third, we studied distancing patterns during different stages of the pandemic to shed light on possible changes over time. Our descriptive results indicated that participants were less likely to physically distance from others in social situations in periods with lower pandemic severity and less stringent measures, indicating that people may less motivated to adhere to distancing guidelines when they perceive the risk of being close to others to be lower, particularly when they are with friends or family [8]. Distancing was also less common in later stages of the pandemic, underscoring the relevance of the severity of the Covid situation as well as duration of the pandemic. However, contrary to our expectations, the difference in distancing behavior between the youngest and older age group was largest in periods with a more severe Covid-19 situation. Given that general adherence to distancing guidelines was also higher during such periods, a likely explanation is that older age groups more strongly increased their adherence to distancing guidelines when the severity of the Covid pandemic increased, while younger participants, for whom Covid was less dangerous, increased their adherence less strongly [4, 25]. This translated in larger differences in distancing behavior compared with more lenient periods, when people of all ages were relatively less likely to adhere to distancing guidelines.

### Limitations and Suggestions for Future Research

In this study, we were able to investigate participants’ physical distancing behavior across various situations, in line with the idea that some people are more motivated to keep their distance than others and that some situations provide better opportunities to physically distance than others. For situations such as the work environment, we were able to measure differences in the opportunity to distance, but for other situations, opportunities, and restrictions for physical distancing had to be inferred (e.g., difficultly to distance when grocery shopping). Similarly, the found differences in participants’ general distancing behavior indicated that people differ in their general capabilities and motivations to distance from others, but these factors were also not measured directly. We therefore suggest future research to investigate people’s perceived opportunity, capability, and motivation for physical distancing in different situations. Nevertheless, and importantly, our study highlights the need to account for situational differences when studying physical distancing behavior, particularly differences in physical context and social relations.

A second limitation is that our Latent Class Analyses resulted in models with relatively low entropy, indicating that classes may have not been separated well. This could be because classes mostly differed in general adherence to distancing guidelines, which is a gradual distinction rather than a categorical one. When differences in general distancing behavior are gradual, distinguishing classes with low, moderate, and high adherence will be based on arbitrary cutoff points and thus possible overlap between classes.

Finally, although we studied different phases of the pandemic and found some variation over time, we could only attribute these to general differences between these phases and not to specific characteristics of these phases. The relatively lower adherence in the latest round in our study (winter 2022), relative to other rounds with high infections and stringent policies, could for instance be due to the either relatively lower perceived Covid risk (due to, for example, the high vaccination rate in the Netherlands), pandemic fatigue due to the long duration of the pandemic, or (a combination of) other factors. We therefore suggest future research to study how contextual factors affect distancing behavior in different situations as well as among sociodemographic groups, using data from a larger number of contexts.

### Conclusion and Implications

Across situations and time, people mainly differed in general distancing behavior: some people distance more than others regardless of the context. There were, however, also relevant differences between situations, which could largely be attributed to the physical opportunities contexts provided to distance, which mostly had a similar impact across groups. Most sociodemographic differences in distancing behavior were small, and those that were found could largely be attributed to differences in the opportunities to distance contexts provided to specific groups (e.g., differences between work contexts). This means that improving opportunities to physically distance in particular situations, such as one-way traffic in supermarkets or optimizing distancing in workplaces, could improve distancing behavior among a large share of the population. Alternatively, when such changes are not possible, providing personal protective measures such as face masks in these situations with fewer opportunities to distance could help reduce infection risks.

The between-individual differences in general distancing behavior and the lower adherence to distancing guidelines with friends or family suggest that person-level factors not included in this study directly, such as perceived risk and motivation, also play a key role in physical distancing behavior. Also, people were less likely to adhere to distancing guidelines during periods with fewer Covid hospitalizations, likely because they perceived being close to others as less risky, underscoring the relevance of communicating the reason for behavioral guidelines even during more lenient periods of the pandemic. Moreover, especially for younger people, the importance of social contact for their development and well-being may surpass their motivation to adhere to distancing guidelines [4, 13, 26]. For future pandemic preparedness, it is therefore also key to accommodate young people to safely meet even in times of high pandemic severity or lockdowns, for instance by ensuring easy access to reliable (self-)tests and venues to meet safely.

## Supplementary Material

kaad053_suppl_Supplementary_Appendix

## Data Availability

Data were collected by the Dutch National Institute for Public Health and the Environment and are not publicly available. The authors do not have permission to share the data.
