# Supplementary material for: Context Matters: Patterns in Physical Distancing Behavior Across Situations and Over Time During the Covid-19 Pandemic in the Netherlands
Source: Ann Behav Med. 2023 Sep 14;58(2):79–91. doi: 10.1093/abm/kaad053 (PMC10831219; doi:10.1093/abm/kaad053)
Supplement: kaad053_suppl_Supplementary_Appendix [file kaad053_suppl_supplementary_appendix.docx]

| **Appendix A: Sociodemographic sample characteristics** | | | | | | | | | | | | | | |
| --- | --- | --- | --- | --- | --- | --- | --- | --- | --- | --- | --- | --- | --- | --- |
|  | Round 2 | |  | Round 5 | |  | Round 11 | |  | Round 16 | |  | Round 18 | |
|  | N=52097 | |  | N=46844 | |  | N=47227 | |  | N=38430 | |  | N=44198 | |
|  | N | % |  | N | % |  | N | % |  | N | % |  | N | % |
| Sex |  |  |  |  |  |  |  |  |  |  |  |  |  |  |
| Male | 19991 | 38.37 |  | 18481 | 39.45 |  | 17694 | 37.47 |  | 14648 | 38.14 |  | 16244 | 36.75 |
| Female | 32106 | 61.63 |  | 28363 | 60.55 |  | 29533 | 62.53 |  | 23728 | 61.79 |  | 27954 | 63.25 |
| Other | 0 | 0 |  | 0 | 0 |  | 0 | 0 |  | 27 | 0.07 |  | 0 | 0 |
| Age |  |  |  |  |  |  |  |  |  |  |  |  |  |  |
| 70+ | 9502 | 18.24 |  | 9765 | 20.85 |  | 9550 | 20.22 |  | 9410 | 24.50 |  | 10128 | 22.92 |
| 55-69 | 17553 | 33.69 |  | 17029 | 36.35 |  | 16706 | 35.37 |  | 15101 | 39.32 |  | 16494 | 37.32 |
| 40-54 | 15822 | 30.37 |  | 13375 | 28.55 |  | 12782 | 27.07 |  | 9631 | 25.08 |  | 11805 | 26.71 |
| 25-39 | 8345 | 16.02 |  | 6092 | 13.00 |  | 7253 | 15.36 |  | 3894 | 10.14 |  | 5264 | 11.91 |
| 16-24 | 875 | 1.68 |  | 583 | 1.24 |  | 936 | 1.98 |  | 367 | 0.96 |  | 507 | 1.15 |
| Education level |  |  |  |  |  |  |  |  |  |  |  |  |  |  |
| Low (including no education) | 6303 | 12.10 |  | 5800 | 12.38 |  | 5323 | 11.27 |  | 4618 | 12.03 |  | 4998 | 11.31 |
| Middle | 14261 | 27.37 |  | 12384 | 26.44 |  | 12588 | 26.65 |  | 10160 | 26.46 |  | 11719 | 26.51 |
| High | 31533 | 60.53 |  | 28172 | 60.14 |  | 28770 | 60.92 |  | 23160 | 60.31 |  | 26975 | 61.03 |
| Unknown | 0 | 0 |  | 0 | 0 |  | 546 | 1.16 |  | 465 | 1.21 |  | 506 | 1.14 |
| Born in |  |  |  |  |  |  |  |  |  |  |  |  |  |  |
| Netherlands | 49595 | 95.20 |  | 44525 | 95.05 |  | 44937 | 95.15 |  | 36646 | 95.42 |  | 42093 | 95.24 |
| Elsewhere | 2502 | 4.80 |  | 2177 | 4.65 |  | 2146 | 4.54 |  | 1653 | 4.30 |  | 2001 | 4.53 |
| Unknown | 0 | 0 |  | 0 | 0 |  | 144 | 0.30 |  | 104 | 0.27 |  | 104 | 0.24 |
| Lives with: partner |  |  |  |  |  |  |  |  |  |  |  |  |  |  |
| No | 14765 | 28.34 |  | 13142 | 28.05 |  | 13334 | 28.23 |  | 10865 | 28.29 |  | 12517 | 28.32 |
| Yes | 37332 | 71.66 |  | 33702 | 71.95 |  | 33893 | 71.77 |  | 27538 | 71.71 |  | 31681 | 71.68 |
| Lives with: child(ren) |  |  |  |  |  |  |  |  |  |  |  |  |  |  |
| No | 36511 | 70.08 |  | 34532 | 73.72 |  | 35078 | 74.28 |  | 30243 | 78.75 |  | 33661 | 76.16 |
| Yes | 15586 | 29.92 |  | 12312 | 26.28 |  | 12149 | 25.72 |  | 8160 | 21.25 |  | 10537 | 23.84 |
| Lives with: other adult(s) |  |  |  |  |  |  |  |  |  |  |  |  |  |  |
| No | 44334 | 85.10 |  | 40125 | 85.66 |  | 40287 | 85.31 |  | 32869 | 85.59 |  | 37615 | 85.11 |
| Yes | 7763 | 14.90 |  | 6719 | 14.34 |  | 6940 | 14.69 |  | 5534 | 14.41 |  | 6583 | 14.89 |
| Underlying medical conditions |  |  |  |  |  |  |  |  |  |  |  |  |  |  |
| No | 39119 | 75.09 |  | 35090 | 74.91 |  | 35707 | 75.61 |  | 28634 | 74.56 |  | 33417 | 75.61 |
| Yes | 12978 | 24.91 |  | 11632 | 24.83 |  | 11520 | 24.39 |  | 9769 | 25.44 |  | 10781 | 24.39 |
| Employment |  |  |  |  |  |  |  |  |  |  |  |  |  |  |
| Not employed | 17529 | 33.65 |  | 17134 | 36.58 |  | 16559 | 35.06 |  | 14839 | 38.64 |  | 15968 | 36.13 |
| Employed, not essential worker/vital sector | 19880 | 38.16 |  | 17238 | 36.80 |  | 19012 | 40.26 |  | 14359 | 37.39 |  | 17042 | 38.56 |
| Employed essential worker/vital sector | 14688 | 28.19 |  | 12472 | 26.62 |  | 11656 | 24.68 |  | 9205 | 23.97 |  | 11188 | 25.31 |
| In education |  |  |  |  |  |  |  |  |  |  |  |  |  |  |
| No | 50540 | 97.01 |  | 45675 | 97.50 |  | 45596 | 96.55 |  | 37462 | 97.55 |  | 42952 | 97.18 |
| Yes | 1557 | 2.99 |  | 1169 | 2.50 |  | 1631 | 3.45 |  | 941 | 2.45 |  | 1246 | 2.82 |
| Urbanization municipality |  |  |  |  |  |  |  |  |  |  |  |  |  |  |
| <500 | 3130 | 6.01 |  | 2765 | 5.90 |  | 2881 | 6.10 |  | 2393 | 6.23 |  | 2709 | 6.13 |
| 500-1000 | 9493 | 18.22 |  | 8442 | 18.02 |  | 8409 | 17.81 |  | 6692 | 17.43 |  | 7768 | 17.58 |
| 1000-1500 | 7690 | 14.76 |  | 6831 | 14.58 |  | 6883 | 14.57 |  | 5831 | 15.18 |  | 6755 | 15.28 |
| 1500-2500 | 14962 | 28.72 |  | 14245 | 30.41 |  | 14850 | 31.44 |  | 11776 | 30.66 |  | 13649 | 30.88 |
| 2500 | 16822 | 32.29 |  | 14534 | 31.03 |  | 14184 | 30.03 |  | 11693 | 30.45 |  | 13307 | 30.11 |
| Unkown | 0 | 0 |  | 27 | 0.06 |  | 20 | 0.04 |  | 18 | 0.05 |  | 10 | 0.02 |
| Socioeconomic status municipality | |  |  |  |  |  |  |  |  |  |  |  |  |  |
| Very low | 5502 | 10.56 |  | 4948 | 10.56 |  | 5119 | 10.84 |  | 4166 | 10.85 |  | 4733 | 10.71 |
| Low | 13142 | 25.23 |  | 11690 | 24.96 |  | 10804 | 22.88 |  | 8587 | 22.36 |  | 9820 | 22.22 |
| Intermediate-low | 7167 | 13.76 |  | 6523 | 13.92 |  | 6577 | 13.93 |  | 5173 | 13.47 |  | 6078 | 13.75 |
| Intermediate-high | 11106 | 21.32 |  | 9969 | 21.28 |  | 10691 | 22.64 |  | 8967 | 23.35 |  | 10289 | 23.28 |
| High | 10963 | 21.04 |  | 9940 | 21.22 |  | 10112 | 21.41 |  | 8171 | 21.28 |  | 9510 | 21.52 |
| Very high | 4217 | 8.09 |  | 3712 | 7.92 |  | 3842 | 8.14 |  | 3274 | 8.53 |  | 3705 | 8.38 |
| Unkown | 0 | 0 |  | 62 | 0.13 |  | 82 | 0.17 |  | 65 | 0.17 |  | 63 | 0.14 |

| **Appendix B: Descriptive statistics of physical distancing behaviour in different settings across rounds** | | | | | | | | | | | | | | | | | | | |
| --- | --- | --- | --- | --- | --- | --- | --- | --- | --- | --- | --- | --- | --- | --- | --- | --- | --- | --- | --- |
|  | Round 2 | | |  | Round 5 | | |  | Round 11 | | |  | Round 16 | | |  | Round 18 | | |
|  | N=52097 | | |  | N=46844 | | |  | N=47227 | | |  | N=38403 | | |  | N=44198 | | |
|  | N | % | % in sit. |  | N | % | % in sit. |  | N | % | % in sit. |  | N | % | % in sit. |  | N | % | % in sit. |
| Crowded place |  |  |  |  |  |  |  |  |  |  |  |  |  |  |  |  |  |  |  |
| Never |  |  |  |  |  |  |  |  | 31373 | 66.43 | 66.66 |  | 17155 | 44.67 | 45.17 |  | 27037 | 61.17 | 61.58 |
| Infrequently |  |  |  |  |  |  |  |  | 9454 | 20.02 | 20.09 |  | 12053 | 31.39 | 31.74 |  | 9807 | 22.19 | 22.34 |
| Frequently |  |  |  |  |  |  |  |  | 6236 | 13.20 | 13.25 |  | 8772 | 22.84 | 23.09 |  | 7065 | 15.98 | 16.09 |
| Missing |  |  |  |  |  |  |  |  | 164 | 0.35 |  |  | 423 | 1.10 |  |  | 289 | 0.65 |  |
| Work |  |  |  |  |  |  |  |  |  |  |  |  |  |  |  |  |  |  |  |
| Not in situation | 32346 | 62.09 |  |  | 23759 | 50.72 |  |  | 26017 | 55.09 |  |  | 19804 | 51.57 |  |  | 24299 | 54.98 |  |
| Never closer than 1.5m | 2460 | 4.72 | 12.75 |  | 1788 | 3.82 | 7.95 |  | 2163 | 4.58 | 10.36 |  | 919 | 2.39 | 5.00 |  | 1640 | 3.71 | 8.32 |
| Infrequently closer than 1.5m | 8669 | 16.64 | 44.96 |  | 9010 | 19.23 | 40.02 |  | 10040 | 21.26 | 48.09 |  | 6938 | 18.07 | 37.78 |  | 8944 | 20.24 | 45.41 |
| Frequently closer than 1.5m | 8153 | 15.65 | 42.29 |  | 11712 | 25.00 | 52.03 |  | 8675 | 18.37 | 41.55 |  | 10509 | 27.37 | 57.22 |  | 9114 | 20.62 | 46.26 |
| Missing | 469 | 0.90 |  |  | 575 | 1.23 |  |  | 332 | 0.70 |  |  | 233 | 0.61 |  |  | 201 | 0.45 |  |
| Education |  |  |  |  |  |  |  |  |  |  |  |  |  |  |  |  |  |  |  |
| Not in situation |  |  |  |  | 44671 | 95.36 |  |  | 45364 | 96.06 |  |  | 36050 | 93.87 |  |  | 42025 | 95.08 |  |
| Never closer than 1.5m |  |  |  |  | 525 | 1.12 | 24.40 |  | 485 | 1.03 | 26.34 |  | 420 | 1.09 | 18.11 |  | 572 | 1.29 | 26.54 |
| Infrequently closer than 1.5m |  |  |  |  | 839 | 1.79 | 39.00 |  | 766 | 1.62 | 41.43 |  | 722 | 1.88 | 31.23 |  | 789 | 1.79 | 36.83 |
| Frequently closer than 1.5m |  |  |  |  | 787 | 1.68 | 36.60 |  | 596 | 1.26 | 32.23 |  | 1172 | 3.05 | 50.66 |  | 787 | 1.78 | 36.63 |
| Missing |  |  |  |  | 22 | 0.05 |  |  | 16 | 0.03 |  |  | 39 | 0.10 |  |  | 25 | 0.06 |  |
| Grocery shopping |  |  |  |  |  |  |  |  |  |  |  |  |  |  |  |  |  |  |  |
| Not in situation | 5180 | 9.94 |  |  | 2859 | 6.10 |  |  | 4395 | 9.31 |  |  | 2091 | 5.44 |  |  | 3428 | 7.76 |  |
| Never closer than 1.5m | 1409 | 2.70 | 3.01 |  | 881 | 1.88 | 2.02 |  | 1758 | 3.72 | 4.12 |  | 914 | 2.38 | 2.54 |  | 1481 | 3.35 | 3.66 |
| Infrequently closer than 1.5m | 23477 | 45.06 | 50.25 |  | 17080 | 36.46 | 39.09 |  | 21838 | 46.24 | 51.16 |  | 16214 | 42.22 | 44.99 |  | 20594 | 46.59 | 50.84 |
| Frequently closer than 1.5m | 21840 | 41.92 | 46.74 |  | 25738 | 54.94 | 58.90 |  | 19093 | 40.43 | 44.73 |  | 18912 | 49.25 | 52.48 |  | 18430 | 41.70 | 45.50 |
| Missing | 191 | 0.37 |  |  | 286 | 0.61 |  |  | 143 | 0.30 |  |  | 272 | 0.71 |  |  | 265 | 0.60 |  |
| Visiting with friends or family (1) |  |  |  |  |  |  |  |  |  |  |  |  |  |  |  |  |  |  |  |
| Not in situation | 22145 | 42.51 |  |  | 12622 | 26.94 |  |  |  |  |  |  |  |  |  |  |  |  |  |
| Never closer than 1.5m | 5234 | 10.05 | 17.51 |  | 2787 | 5.95 | 8.16 |  |  |  |  |  |  |  |  |  |  |  |  |
| Infrequently closer than 1.5m | 18213 | 34.96 | 60.90 |  | 17487 | 37.33 | 51.20 |  |  |  |  |  |  |  |  |  |  |  |  |
| Frequently closer than 1.5m | 6461 | 12.40 | 21.60 |  | 13880 | 29.63 | 40.64 |  |  |  |  |  |  |  |  |  |  |  |  |
| Missing | 44 | 0.08 |  |  | 68 | 0.15 |  |  |  |  |  |  |  |  |  |  |  |  |  |
| Visiting with friends or family (2) |  |  |  |  |  |  |  |  |  |  |  |  |  |  |  |  |  |  |  |
| Not in situation | 20593 | 39.53 |  |  | 15740 | 33.60 |  |  |  |  |  |  |  |  |  |  |  |  |  |
| Never closer than 1.5m | 7120 | 13.67 | 22.66 |  | 3764 | 8.04 | 12.15 |  |  |  |  |  |  |  |  |  |  |  |  |
| Infrequently closer than 1.5m | 19135 | 36.73 | 60.89 |  | 18196 | 38.84 | 58.68 |  |  |  |  |  |  |  |  |  |  |  |  |
| Frequently closer than 1.5m | 5168 | 9.92 | 16.45 |  | 9046 | 19.31 | 29.17 |  |  |  |  |  |  |  |  |  |  |  |  |
| Missing | 81 | 0.16 |  |  | 98 | 0.21 |  |  |  |  |  |  |  |  |  |  |  |  |  |
| Visiting with friends or family (3) |  |  |  |  |  |  |  |  |  |  |  |  |  |  |  |  |  |  |  |
| Not in situation |  |  |  |  |  |  |  |  | 9003 | 19.06 |  |  | 4830 | 12.58 |  |  | 7846 | 17.75 |  |
| Never closer than 1.5m |  |  |  |  |  |  |  |  | 6415 | 13.58 | 16.78 |  | 1540 | 4.01 | 6.05 |  | 2798 | 6.33 | 10.15 |
| Infrequently closer than 1.5m |  |  |  |  |  |  |  |  | 24158 | 51.15 | 63.21 |  | 12532 | 32.63 | 49.22 |  | 15876 | 35.92 | 57.61 |
| Frequently closer than 1.5m |  |  |  |  |  |  |  |  | 7648 | 16.19 | 20.01 |  | 11389 | 29.66 | 44.74 |  | 8886 | 20.10 | 32.24 |
| Missing |  |  |  |  |  |  |  |  | 3 | 0.01 |  |  | 8112 | 21.12 |  |  | 8792 | 19.89 |  |
| Outdoors (1) |  |  |  |  |  |  |  |  |  |  |  |  |  |  |  |  |  |  |  |
| Not in situation | 35026 | 67.23 |  |  | 32968 | 70.38 |  |  |  |  |  |  |  |  |  |  |  |  |  |
| Never closer than 1.5m | 6366 | 12.22 | 37.62 |  | 4881 | 10.42 | 35.42 |  |  |  |  |  |  |  |  |  |  |  |  |
| Infrequently closer than 1.5m | 8957 | 17.19 | 52.92 |  | 7305 | 15.59 | 52.99 |  |  |  |  |  |  |  |  |  |  |  |  |
| Frequently closer than 1.5m | 1599 | 3.07 | 9.45 |  | 1596 | 3.41 | 11.59 |  |  |  |  |  |  |  |  |  |  |  |  |
| Missing | 149 | 0.29 |  |  | 94 | 0.20 |  |  |  |  |  |  |  |  |  |  |  |  |  |
| Outdoors (2) |  |  |  |  |  |  |  |  |  |  |  |  |  |  |  |  |  |  |  |
| Not in situation | 10434 | 20.03 |  |  | 10247 | 21.87 |  |  |  |  |  |  |  |  |  |  |  |  |  |
| Never closer than 1.5m | 11489 | 22.05 | 27.63 |  | 10278 | 21.94 | 28.12 |  |  |  |  |  |  |  |  |  |  |  |  |
| Infrequently closer than 1.5m | 25143 | 48.26 | 60.48 |  | 21649 | 46.22 | 59.25 |  |  |  |  |  |  |  |  |  |  |  |  |
| Frequently closer than 1.5m | 4943 | 9.49 | 11.89 |  | 4612 | 9.85 | 12.63 |  |  |  |  |  |  |  |  |  |  |  |  |
| Missing | 88 | 0.17 |  |  | 58 | 0.12 |  |  |  |  |  |  |  |  |  |  |  |  |  |
| Outdoors (3) |  |  |  |  |  |  |  |  |  |  |  |  |  |  |  |  |  |  |  |
| Not in situation |  |  |  |  |  |  |  |  | 4028 | 8.53 |  |  | 4072 | 10.60 |  |  | 4661 | 10.55 |  |
| Never closer than 1.5m |  |  |  |  |  |  |  |  | 15132 | 32.04 | 35.15 |  | 11698 | 30.46 | 34.29 |  | 17514 | 39.63 | 44.55 |
| Infrequently closer than 1.5m |  |  |  |  |  |  |  |  | 24190 | 51.22 | 56.19 |  | 19340 | 50.36 | 56.70 |  | 19480 | 44.07 | 49.54 |
| Frequently closer than 1.5m |  |  |  |  |  |  |  |  | 3727 | 7.89 | 8.66 |  | 3073 | 8.00 | 9.01 |  | 2319 | 5.25 | 5.90 |
| Missing |  |  |  |  |  |  |  |  | 150 | 0.32 |  |  | 220 | 0.57 |  |  | 224 | 0.51 |  |
| Informal care |  |  |  |  |  |  |  |  |  |  |  |  |  |  |  |  |  |  |  |
| Not in situation | 40547 | 77.83 |  |  | 37366 | 79.77 |  |  | 37332 | 79.05 |  |  | 30464 | 79.33 |  |  | 34815 | 78.77 |  |
| Never closer than 1.5m | 3667 | 7.04 | 31.88 |  | 1502 | 3.21 | 15.95 |  | 1798 | 3.81 | 18.25 |  | 858 | 2.23 | 10.86 |  | 1625 | 3.68 | 17.42 |
| Infrequently closer than 1.5m | 5711 | 10.96 | 49.64 |  | 4599 | 9.82 | 48.81 |  | 5182 | 10.97 | 52.54 |  | 3554 | 9.25 | 45.06 |  | 4401 | 9.96 | 47.14 |
| Frequently closer than 1.5m | 2127 | 4.08 | 18.48 |  | 3319 | 7.09 | 35.24 |  | 2880 | 6.10 | 29.21 |  | 3477 | 9.05 | 44.08 |  | 3310 | 7.49 | 35.45 |
| Missing | 45 | 0.09 |  |  | 58 | 0.12 |  |  | 35 | 0.07 |  |  | 50 | 0.13 |  |  | 47 | 0.11 |  |
| Formal care |  |  |  |  |  |  |  |  |  |  |  |  |  |  |  |  |  |  |  |
| Not in situation | 40818 | 78.35 |  |  |  |  |  |  |  |  |  |  |  |  |  |  |  |  |  |
| Never closer than 1.5m | 6570 | 12.61 | 58.30 |  |  |  |  |  |  |  |  |  |  |  |  |  |  |  |  |
| Infrequently closer than 1.5m | 4004 | 7.69 | 35.55 |  |  |  |  |  |  |  |  |  |  |  |  |  |  |  |  |
| Frequently closer than 1.5m | 693 | 1.33 | 6.15 |  |  |  |  |  |  |  |  |  |  |  |  |  |  |  |  |
| Missing | 12 | 0.02 |  |  |  |  |  |  |  |  |  |  |  |  |  |  |  |  |  |
| Hotels, bars and restaurants |  |  |  |  |  |  |  |  |  |  |  |  |  |  |  |  |  |  |  |
| Not in situation |  |  |  |  | 26561 | 56.70 |  |  |  |  |  |  | 17932 | 46.69 |  |  |  |  |  |
| Never closer than 1.5m |  |  |  |  | 2618 | 5.59 | 12.92 |  |  |  |  |  | 1392 | 3.62 | 6.82 |  |  |  |  |
| Infrequently closer than 1.5m |  |  |  |  | 12558 | 26.81 | 61.97 |  |  |  |  |  | 10663 | 27.77 | 52.33 |  |  |  |  |
| Frequently closer than 1.5m |  |  |  |  | 5087 | 10.86 | 25.10 |  |  |  |  |  | 8327 | 21.68 | 40.85 |  |  |  |  |
| Missing |  |  |  |  | 20 | 0.04 |  |  |  |  |  |  | 89 | 0.23 |  |  |  |  |  |
| Cultural venues |  |  |  |  |  |  |  |  |  |  |  |  |  |  |  |  |  |  |  |
| Not in situation |  |  |  |  | 43153 | 92.12 |  |  |  |  |  |  | 30385 | 79.12 |  |  |  |  |  |
| Never closer than 1.5m |  |  |  |  | 933 | 1.99 | 25.25 |  |  |  |  |  | 739 | 1.92 | 9.22 |  |  |  |  |
| Infrequently closer than 1.5m |  |  |  |  | 2245 | 4.79 | 60.79 |  |  |  |  |  | 3367 | 8.77 | 42.12 |  |  |  |  |
| Frequently closer than 1.5m |  |  |  |  | 513 | 1.10 | 13.96 |  |  |  |  |  | 3891 | 10.13 | 48.66 |  |  |  |  |
| Missing |  |  |  |  | 0.00 | 0.00 |  |  |  |  |  |  | 21 | 0.05 |  |  |  |  |  |
| Sport |  |  |  |  |  |  |  |  |  |  |  |  |  |  |  |  |  |  |  |
| Not in situation |  |  |  |  | 35947 | 76.74 |  |  | 41066 | 86.95 |  |  | 26393 | 68.73 |  |  | 33535 | 75.87 |  |
| Never closer than 1.5m |  |  |  |  | 1670 | 3.57 | 15.35 |  | 1485 | 3.14 | 24.14 |  | 1212 | 3.16 | 10.15 |  | 1694 | 3.83 | 15.93 |
| Infrequently closer than 1.5m |  |  |  |  | 6559 | 14.00 | 60.22 |  | 3876 | 8.21 | 63.11 |  | 6480 | 16.87 | 54.17 |  | 6518 | 14.75 | 61.36 |
| Frequently closer than 1.5m |  |  |  |  | 2661 | 5.68 | 24.43 |  | 783 | 1.66 | 12.76 |  | 4267 | 11.11 | 35.68 |  | 2414 | 5.46 | 22.71 |
| Missing |  |  |  |  | 7 | 0.01 |  |  | 17 | 0.04 |  |  | 51 | 0.13 |  |  | 37 | 0.08 |  |
| Party |  |  |  |  |  |  |  |  |  |  |  |  |  |  |  |  |  |  |  |
| Not in situation |  |  |  |  |  |  |  |  | 45058 | 95.41 |  |  | 29914 | 77.89 |  |  | 40531 | 91.70 |  |
| Never closer than 1.5m |  |  |  |  |  |  |  |  | 385 | 0.82 | 17.90 |  | 393 | 1.02 | 4.62 |  | 350 | 0.79 | 9.54 |
| Infrequently closer than 1.5m |  |  |  |  |  |  |  |  | 1076 | 2.28 | 49.78 |  | 2943 | 7.66 | 34.71 |  | 1569 | 3.55 | 42.87 |
| Frequently closer than 1.5m |  |  |  |  |  |  |  |  | 701 | 1.48 | 32.31 |  | 5141 | 13.39 | 60.67 |  | 1740 | 3.94 | 47.58 |
| Missing |  |  |  |  |  |  |  |  | 7 | 0.01 |  |  | 12 | 0.03 |  |  | 8 | 0.02 |  |

**Appendix C: Latent Class Analyses**

*Round 2*

Table C1 presents the results of the LCA of data collected in round 2, with solutions up to 20 latent classes, while Figure C1 provides the change in model fit with each additional class. In terms of model fit, the best solution was found with 16 (lowest BIC) or even more (AIC) classes.^[[1]](#footnote-1)^ However, compared to models with fewer classes, the improvement in model fit was already small for models with 5 and 6 classes and marginal for models with 7 classes or more. Due to the very large N in our analysis, distinguishing large numbers of classes thus led to a significant, but not a substantial improvement in model fit. We therefore considered the models with 5 and 6 classes as optimal in terms of model fit.

In terms of interpretation, the second criterium, models with 2 to 4 classes distinguished clusters based on their general adherence to distancing guidelines. With 5 to 7 classes, situational differences emerged: first only based on distancing from friends or family (5 classes), then also at work (6 classes) and outdoors and when providing informal care (7 classes). Models with 8 or more classes further separated classes according to general adherence and/or these specific situations. Given that these models did not substantially improve model fit, we did not describe these classes in detail.

Entropy of all models is low (<0.6) indicating ‘fuzziness’ or poor separation of classes. This means we cannot select an optimal model for round 2. However, overall, the models indicate that participants mainly differed with respect to general adherence. Moreover, the models with reasonable model fit (the models with 5 and 6 classes) indicate that people may also exhibit more specific behavioural patterns with friends or family and at work.

**Figure C1: Change in model fit LCA round 2**

| **Table C1: LCA Model fit information Round 2** | | | | |
| --- | --- | --- | --- | --- |
| **Model** | **BIC** | **AIC** | **Entropy** | **Class description (in terms of violations of distancing)** |
| Model 1 | 862123.5 | 861910.4 |  |  |
| Model 2 | 845442.8 | 845007.8 | 0.49 | Differences in adherence (general, not situation-specific):  • Low (58%) • High (42%) |
| Model 3 | 837403.9 | 836746.9 | 0.54 | Differences in adherence (general, not situation-specific):  • Low (42%) • Intermediate (31%) • High (27%) |
| Model 4 | 832846.4 | 831967.6 | 0.56 | Differences in adherence (general, not situation-specific):  • Low (27%) • Intermediate: low mobility (28%) • Intermediate: higher mobility (30%) • High (15%) |
| Model 5 | 830015.4 | 828914.6 | 0.56 | Differences in adherence and specifically distancing from friends or family:  • Low (25%) • Intermediate-low: higher mobility, but often distance (14%) • Intermediate: low mobility (infrequently visits friends or family), mixed on distancing (21%) • Intermediate: general (29%) • High (11%) |
| Model 6 | 828191.4 | 826868.7 | 0.57 | Differences in adherence and specifically distancing from friends or family and at work:  • Low (18%)  • Intermediate-low: somewhat less distancing with friends or family and at work (11%) • Intermediate: low mobility (infrequently visits friends or family), moderate on distancing (17%)  • Intermediate: general (28%)  • Intermediate-high: similar to high-violations clusters, but more distancing from friends or family (16%)  • High (9%) |
| Model 7 | 827110.9 | 825566.3 | 0.57 | Differences in adherence and specifically distancing from friends or family and at work, as well as outdoors and when providing informal care:  • Low (16%)  • Intermediate-low with somewhat lower mobility: somewhat less distancing with friends or family and at work (17%)  • Intermediate-low with somewhat higher mobility: somewhat less distancing with friends or family and at work (7%) • Intermediate: general, somewhat more distancing outdoors and when providing informal care (12%) • Intermediate: general, somewhat less distancing outdoors and when providing informal care (30%) • Intermediate-high: similar to high-violations clusters, but more distancing from friends or family (9%)  • High (9%) |
| Model 8 | 826140.9 | 824374.3 | 0.57 |  |
| Model 9 | 825506.9 | 823518.4 | 0.56 |  |
| Model 10 | 824927.6 | 822717.2 | 0.56 |  |
| Model 11 | 824595.2 | 822162.9 | 0.57 |  |
| Model 12 | 824246 | 821591.8 | 0.56 |  |
| Model 13 | 824102.6 | 821226.4 | 0.56 |  |
| Model 14 | 823961.5 | 820863.4 | 0.54 |  |
| Model 15 | 823857.2 | 820537.1 | 0.53 |  |
| Model 16 | 823844.3 | 820302.3 | 0.54 |  |
| Model 17 | 823912 | 820148.1 | 0.54 |  |

*Round 5*

Table C2 presents the results of the LCA of round 5 and Figure C2 the changes in model fit. Again, the best model fit was found for models with very high numbers of classes, namely 17 (lowest BIC) or more (lower AIC) classes. However, the improvement in model fit reduced substantially the more classes were added: it was large with 2 and 3 classes, moderate for 4-6, small for 7 and 8 classes and marginal with 9 or more classes. We therefore considered models with 4 to 8 classes optimal in terms of model fit.

Models with up to 3 classes only distinguished clusters based on general distancing behaviour, while situation-specific distinctions were added when more classes were estimated. Models with 4 and 5 classes distinguished classes based on general adherence as well as distancing outdoors. With 6 classes, the class with intermediate-high violations was split into two, which differed in distancing behaviour in many settings, but this distinction disappears with 7 to 8 classes. The model with 7 classes differentiated classes with respect to general adherence as well as distancing with friends or family and at work. Finally, the model with 8 classes added distinctions based on distancing outdoors to the 7 class-solution. Models with 9 or more classes further separated classes according to general adherence and/or these specific situations. Given that these models did not substantially improve model fit, we did not describe these classes in detail.

Considering both model fit and substantive interpretation, the model with 8 classes was considered optimal for round 5. It had a small improvement in model fit compared to the model with 7 classes and distinguished classes based on general adherence as well as the three specific situations that were also influential in distinguishing classes in the models with fewer classes: outdoors, with friends or family and at work. Like the other models with up to 9 classes, this model also had relatively low but sufficient entropy (0.61).

**Figure C2: Change in model fit LCA round 5**

| **Table C2: LCA Model fit information Round 5** | | | | |
| --- | --- | --- | --- | --- |
| **Model** | **BIC** | **AIC** | **Entropy** | **Interpretation (violations)** |
| Model 1 | 1011689 | 1011382 |  |  |
| Model 2 | 978685.5 | 978063.9 | 0.65 | Differences in adherence (general, not situation-specific):  • Low (60%) • High (40%) |
| Model 3 | 969832.2 | 968895.4 | 0.60 | Differences in adherence (general, not situation-specific):  • Low (35%) • Intermediate (34%) • High (30%) |
| Model 4 | 965399.6 | 964147.6 | 0.61 | Differences in adherence and specifically distancing outdoors:  • Low: low mobility, not always distance (mainly outdoors) (21%) • Intermediate-low: higher frequency, distances outdoors (20%)  • Intermediate (31%) • High (28%) |
| Model 5 | 962014.8 | 960447.7 | 0.61 | Differences in adherence and specifically distancing outdoors:  • Low: low mobility, not always distance (mainly outdoors) (19%) • Intermediate-low: higher frequency, distances outdoors (15%) • Intermediate: relatively higher mobility than below, but more often distance (27%) • Intermediate-high (19%) • High (20%) |
| Model 6 | 959541.1 | 957658.8 | 0.61 | Differences in adherence and specifically distancing outdoors + multiple other settings:  • Low (17%) • Intermediate-low: higher mobility than above, but more distance outdoors (15%)  • Intermediate (24%)  • Intermediate-high: relatively low mobility, but less often distance (particularly at cultural venues, supermarket and outdoors) (18%)  • Intermediate-high: high mobility, unlikely to keep distance when providing informal care and with friends or family, at organized sport and at work (16%)  • High (9%) |
| Model 7 | 957720.7 | 955523.2 | 0.61 | Differences in adherence and specifically distancing with friends or family and at work:  • Low (12%)  • Intermediate-low (19%)  • Intermediate: relatively low mobility, but less often distance (15%)  • Intermediate: but less likely to keep distance from friends or family and at work (10%)  • Intermediate-high: relatively low mobility, but less often distance (20%)  • Intermediate-high: particularly unlikely to keep distance from friends or family (15%)  • High (8%) |
| Model 8 | 956132.6 | 953619.9 | 0.61 | Differences in adherence and specifically distancing outdoors, with friends or family and at work:  • Low mobility, most likely to keep distance except outdoors and with friends or family (10%)  • Low: somewhat higher mobility, but often distance (most likely outdoors and with friends or family) (8%)  • Intermediate: relatively low mobility, but less often distance (14%)  • Intermediate: but less likely to keep distance from friends or family and at work (15%) • Intermediate-high : relatively low mobility, but less often distance (15%) • Intermediate-high: general (17%) • Intermediate-high: particularly unlikely to keep distance from friends or family (14%) • High (8%) |
| Model 9 | 955413.8 | 952585.9 | 0.60 |  |
| Model 10 | 954886.2 | 951743.2 | 0.59 |  |
| Model 11 | 954475.2 | 951016.9 | 0.58 |  |
| Model 12 | 954167.8 | 950394.4 | 0.57 |  |
| Model 13 | 953889.9 | 949801.4 | 0.58 |  |
| Model 14 | 953721.1 | 949317.4 | 0.58 |  |
| Model 15 | 953559.3 | 948840.4 | 0.55 |  |
| Model 16 | 953488.4 | 948454.3 | 0.55 |  |
| Model 17 | 953473 | 948123.7 | 0.57 |  |
| Model 18 | 953488.5 | 947824 | 0.56 |  |

*Round 11*

The results for round 11 are presented in Table C3 and Figure C3. Regarding model fit, BIC was lowest for the model with 10 classes. However, improvement in model fit reduced substantially the more classes were added: there was a large improvement for a model with 2 classes, intermediate for 3 to 5 classes and it became marginal with 6 classes or more. This indicates that the model with 5 classes was optimal in terms of model fit.

Substantially, the models with 2 and 3 classes distinguished classes based on general adherence. The model with 4 classes also distinguished classes based on distancing outdoors, with friends or family and at work. The model with 5 classes further split the class with immediate-high violations into two: one class who was less likely to keep distance in more informal, social settings (namely with friends or family and when providing informal care) and one class who more often violated distancing guidelines in more formal, regulated settings (the supermarket and in education or at work) and more often visited crowded places. The model with 6 classes added another distinction based on general adherence. Since model fit only marginally improved with 6 classes or more, we did not describe the classes of models with >7 classes in detail.

Considering both model fit and substantive interpretation, model 5 seemed optimal, but this model did not have sufficient entropy (<0.6). Entropy was low, but sufficient for the model with 2 classes, but this model did not have good model fit. We therefore could not select an optimal model for round 11. Overall, the models indicate that participants mostly differed in general distancing behaviour, while model 5 indicates that some people may have had particular difficulty distancing in informal social settings (such as with friends or family) while others may be less likely to adhere to distancing guidelines in more regulated and crowded places (such as the supermarket or at work).

**Figure C3: Change in model fit LCA round 11**

| **Table C3: LCA Model fit information Round 11** | | | | |
| --- | --- | --- | --- | --- |
| **Model** | **BIC** | **AIC** | **Entropy** | **Interpretation (violations)** |
| Model 1 | 664607.5 | 664379.7 |  |  |
| Model 2 | 643865.8 | 643401.3 | 0.62 | Differences in adherence (general, not situation-specific):  • Low (60%) • High (40%) |
| Model 3 | 640327.2 | 639626.2 | 0.52 | Differences in adherence (general, not situation-specific):  • Low (30%) • Intermediate (38%) • High (33%) |
| Model 4 | 637786.4 | 636848.8 | 0.52 | Differences in adherence and specifically distancing outdoors, with friends or family and at work:  • Low(mobility), intermediate outdoors, with friends or family and at work (relative to low mobility) (14%) • Intermediate-low (distances outdoors, also relatively often distances from friends or family and at work) (20%) • Intermediate (35%) • High (32%) |
| Model 5 | 635778.9 | 634604.6 | 0.54 | Differences in adherence and specifically distancing outdoors, with friends or family, at work, when providing informal care and at the supermarket:  • Low(mobility), intermediate outdoors, with friends or family and at work (relative to low mobility) (11%) • Intermediate-low (distances outdoors, also relatively often distances from friends or family and at work) (19%) • Intermediate: relatively less likely to keep distance when providing informal care and with friends or family (31%) • Intermediate-high: relatively less likely to keep distance in supermarket and in education/at work, relatively often visits crowded place (30%) • High (10%) |
| Model 6 | 635220.1 | 633809.2 | 0.56 | Differences in adherence and specifically distancing outdoors, with friends or family, at work and at the supermarket:  • Low(mobility), intermediate outdoors, with visitors and at work (relative to low mobility) (11%) • Intermediate-low (distances outdoors, also relatively often distances from visitors and at work) (18%) • Intermediate (32%) • Intermediate-high: relatively less likely to keep distance in supermarket and in education/at work, relatively often visits crowded places (22%) • Intermediate-high: unlikely to keep distance in education/at work (7%) • High (9%) |
| Model 7 | 634811.6 | 633164.1 | 0.57 |  |
| Model 8 | 634447.7 | 632563.6 | 0.55 |  |
| Model 9 | 634160.6 | 632039.9 | 0.54 |  |
| Model 10 | 634103.8 | 631746.5 | 0.52 |  |
| Model 11 | 634112 | 631518 | 0.56 |  |
| Model 12 | 634172.9 | 631342.3 | 0.54 |  |
| Model 13 | 634264 | 631196.9 | 0.51 |  |
| Model 14 | 634393.2 | 631089.4 | 0.51 |  |
| Model 15 | 634549 | 631008.6 | 0.53 |  |
| Model 16 | 634706.1 | 630929.1 | 0.54 |  |
| Model 17 | 634876.9 | 630863.4 | 0.50 |  |
| Model 18 | 635039.2 | 630789 | 0.49 |  |
| Model 19 | 635207.5 | 630720.7 | 0.46 |  |
| Model 20 | 635396.1 | 630672.6 | 0.42 |  |

*Round 16*

The results for round 16 are presented in Table C4 and Figure C4. Regarding model fit, BIC was lowest for the model with 13 classes and AIC reduced with every additional class. However, improvement in model fit reduced substantially the more classes were added: there was a large improvement for a model with 2 and 3 classes, intermediate for 4 to 6, small for 7 and marginal for models with 8 classes or more. We therefore considered models with 4 to 7 classes optimal in terms of model fit.

Substantially, the models with 2 to 5 classes distinguished classes based on general adherence. With 6 classes, situation-specific distinctions emerged. Similar to round 11, distinctions were based on distancing outdoors, as well as social settings (with friends or family, cultural venues and bars, restaurants and hotels, parties) and more formal regulated settings (such as the supermarket and at work), the latter two distinguish classes with intermediate-high levels of violating distancing guidelines. Models with 7 or 8 classes only differentiated further based on general adherence and the same situations as the model with 6 classes.

Considering both model fit and substantive interpretation, model 6 seemed optimal, but this model did not have sufficient entropy (<0.6). Entropy was low, but sufficient for the models with 2 and 3 classes, but these models did not have good model fit. We therefore could not select an optimal model for round 16. However, similar to round 11, the results of the LCA of round 16 indicate that participants mainly differed in general distancing behaviour, with possible additional distinctions due to behaviour in informal vs. formal settings.

**Figure C4: Change in model fit LCA round 16**

| **Table C4: LCA Model fit information Round 16** | | | | |
| --- | --- | --- | --- | --- |
| **Model** | **BIC** | **AIC** | **Entropy** | **Interpretation (violations)** |
| Model 1 | 740762.5 | 740488.7 |  |  |
| Model 2 | 711071.9 | 710515.8 | 0.67 | Differences in adherence (general, not situation-specific):  • Low (57%) • High (43%) |
| Model 3 | 705093.3 | 704254.8 | 0.60 | Differences in adherence (general, not situation-specific):  • Low (26%) • Intermediate (38%) • High (36%) |
| Model 4 | 702834 | 701713.1 | 0.57 | Differences in adherence (general, not situation-specific):  • Low: high mobility, but often distance (19%) • Intermediate: low mobility, but less often distance (16%)  • Intermediate (36%) • High (28%) |
| Model 5 | 701126.5 | 699723.4 | 0.56 | Differences in adherence (general, not situation-specific):  • Low (16%)  • Intermediate: low mobility, but less often distance (16%)  • Intermediate (17%)  • Intermediate-high: (33%)  • High (17%) |
| Model 6 | 699686.9 | 698001.4 | 0.56 | Differences in adherence and specifically distancing outdoors, at the supermarket, at work, in cultural venues and bars, restaurants and hotels, at parties and with friends or family:  • Low: low mobility, often distance, but somewhat less outdoors (12%)  • Low: high mobility, often distance, somewhat more outdoors (13%) • Intermediate (18%)  • Intermediate-high: particularly less distance in supermarket, outdoors, and at work (20%) • Intermediate-high: particularly less distance in cultural venues, bars, restaurants and hotels, parties and with friends or family (23%)  • High (13%) |
| Model 7 | 698806.6 | 696838.7 | 0.55 | Differences in adherence and specifically distancing outdoors, at the supermarket, at work, in cultural venues and bars, restaurants and hotels, at parties and with friends or family:  • Low: low mobility, often distance, but somewhat less outdoors (9%) • Low: high mobility, often distance, somewhat more outdoors (11%) • Intermediate (18%)  • Intermediate-high: general (18%)  • Intermediate-high: particularly less distance in supermarket and work (13%)  • Intermediate-high: particularly less distance in cultural venues, bars, restaurants and hotels, parties and with friends or family (17%)  • High (15%) |
| Model 8 | 698348.1 | 696097.9 | 0.56 | Differences in adherence and specifically distancing outdoors, at the supermarket, at work, in cultural venues and bars, restaurants and hotels, at parties and with friends or family:  • Low (9%)  • Intermediate-low: higher mobility (11%)  • Intermediate (18%)  • Intermediate mixed: relatively high mobility, but does not often go to parties or visits with friends or family (13%) • Intermediate-high: general (13%)  • Intermediate-high: particularly less distance from friends or family and does not often visit cultural venues (11%) • Intermediate-high: particularly less distance in cultural venues, bars, restaurants and hotels and at, parties, but more distancing in supermarket (15%)  • High (14%) |
| Model 9 | 698141.9 | 695609.3 | 0.56 |  |
| Model 10 | 697966.5 | 695151.6 | 0.53 |  |
| Model 11 | 697890.8 | 694793.5 | 0.55 |  |
| Model 12 | 697815 | 694435.5 | 0.52 |  |
| Model 13 | 697809.5 | 694147.6 | 0.54 |  |
| Model 14 | 697852.3 | 693908.1 | 0.53 |  |
| Model 15 | 697932.3 | 693705.7 | 0.53 |  |
| Model 16 | 698029.8 | 693520.8 | 0.51 |  |
| Model 17 | 698154.2 | 693362.9 | 0.51 |  |
| Model 18 | 698315.9 | 693242.2 | 0.53 |  |
| Model 19 | 698491.4 | 693135.4 | 0.49 |  |
| Model 20 | 698666.5 | 693028.1 | 0.49 |  |

*Round 18*

Table C5 presents the result of the LCA of round 18. Regarding model fit, BIC was lowest for the model with 10 classes and AIC reduces with every additional class. However, again, the improvement in model fit with large numbers of classes became marginal with higher numbers of classes. There was a large improvement in model fit for the model with 2 classes, models with 3 to 5 classes had a moderate improvement in fit and with 6 or more classes there was only a marginal improvement. We therefore considered the models with 3 to 5 classes as having optimal model fit.

Substantially, the models with 2 and 3 classes only distinguished classes with respect to general distancing behaviour, while the model with 4 classes added a distinction based on distancing outdoors. The model with 5 classes added another distinction based on general distancing. The models with 6 and 7 classes added further situation-specific distinctions similar to those found in round 11 and 16 (based on more informal, social settings and formal, regulated settings). From model 8 onwards, only further distinctions are made based on general adherence and these settings. Given the small improvement in model fit, these classes are not discussed in detail.

Considering both model fit and substantive interpretation, model 4 seemed optimal, but this model did not have sufficient entropy (<0.6). Entropy was low, but sufficient for the model with 2 classes, but this model did not have good model fit. We therefore could not select an optimal model for round 18. Overall, however, the LCA indicates that participants mainly differed in general adherence and that situation-specific differences seemed mainly related to distancing outdoors in round 18.

**Figure C5: Change in model fit LCA round 18**

| **Table C5: LCA Model fit information Round 18** | | | | |
| --- | --- | --- | --- | --- |
| **Model** | **BIC** | **AIC** | **Entropy** | **Interpretation (violations)** |
| Model 1 | 643335.2 | 643109.1 |  |  |
| Model 2 | 620799.9 | 620339 | 0.63 | Differences in adherence (general, not situation-specific):  • Low (60%) • High (40%) |
| Model 3 | 616851.9 | 616156.1 | 0.55 | Differences in adherence (general, not situation-specific):  • Low (22%) • Intermediate (44%) • High (34%) |
| Model 4 | 614568.1 | 613637.5 | 0.53 | Differences in adherence and specifically distancing outdoors:  • Low: low mobility, intermediate on distancing outdoors (10%) • Intermediate-low: distances outdoors (17%) • Intermediate-high (41%) • High (32%) |
| Model 5 | 612565.9 | 611400.5 | 0.54 | Differences in adherence and specifically distancing outdoors  • Low: low mobility, intermediate on distancing outdoors (8%)  • Intermediate-low: distances outdoors (15%) • Intermediate (30%) • Intermediate-high (34%) • High (13%) |
| Model 6 | 611896.9 | 610496.6 | 0.57 | Differences in adherence and specifically distancing outdoors, and at work or in education  • Low: low mobility, intermediate on distancing outdoors (10%) • Intermediate-low: distances outdoors (15%) • Intermediate (31%) • Intermediate-high (25%) • Intermediate-high: particularly less likely to distance at work and in education (7%) • High (11%) |
| Model 7 | 611313.4 | 609678.3 | 0.55 | Differences in adherence and specifically distancing outdoors, at work or in education and social settings  • Low: low mobility, intermediate on distancing outdoors (10%) • Intermediate-low: distances outdoors (15%) • Intermediate-low: general (28%) • Intermediate: general (22%)  • Intermediate-high: particularly less likely to distance when providing informal care, at parties and with friends or family (9%) • Intermediate-high: particularly less likely to distance at work and in education (7%) • High (9%) |
| Model 8 | 610887.7 | 609017.8 | 0.54 |  |
| Model 9 | 610740.9 | 608636.2 | 0.53 |  |
| Model 10 | 610572.8 | 608233.2 | 0.52 |  |
| Model 11 | 610578.6 | 608004.3 | 0.53 |  |
| Model 12 | 610625 | 607815.8 | 0.51 |  |
| Model 13 | 610718 | 607673.7 | 0.50 |  |
| Model 14 | 610823 | 607544.3 | 0.50 |  |
| Model 15 | 610959 | 607445 | 0.49 |  |
| Model 16 | 611096 | 607347.9 | 0.48 |  |
| Model 17 | 611235 | 607252 | 0.48 |  |
| Model 18 | 611400 | 607182.4 | 0.47 |  |
| Model 19 | 611584 | 607130.8 | 0.46 |  |
| Model 20 | 611736 | 607047.8 | 0.45 |  |

**Appendix D: Associations between sociodemographic characteristics and situation-specific distancing behaviour**

**
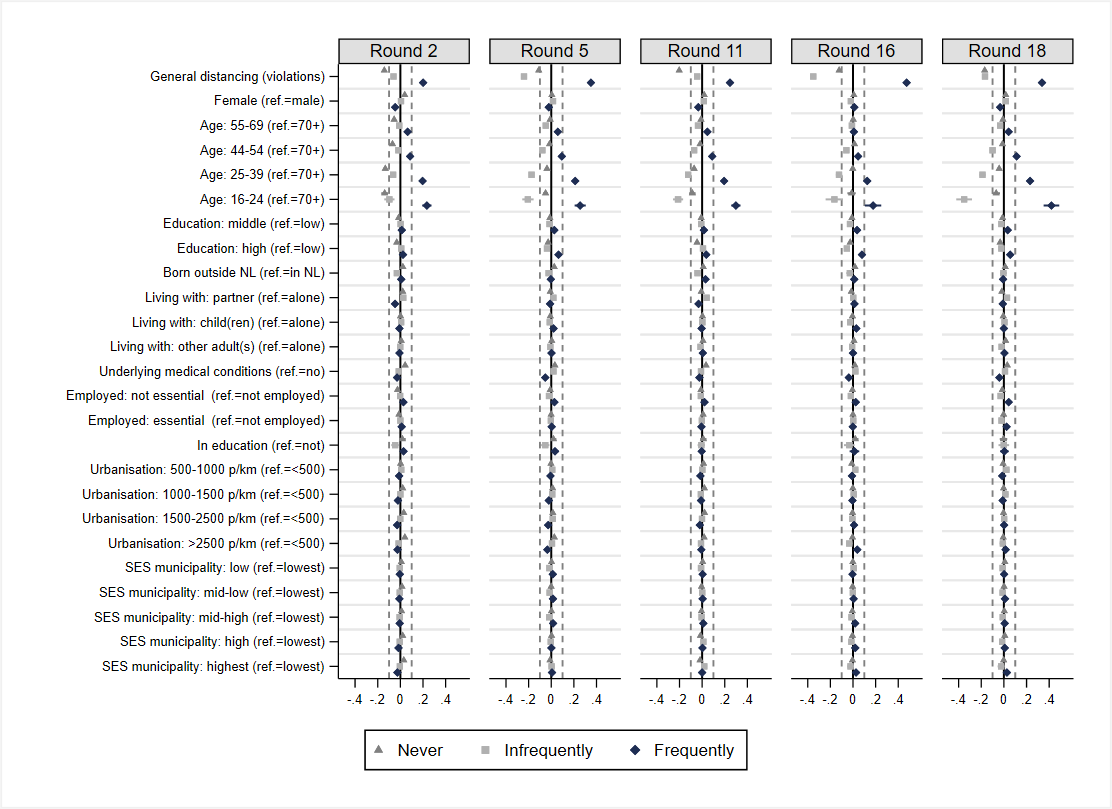
**

**Figure D1: Associations between sociodemographic factors and physical distancing when visiting with friends or family (average marginal effects)**

**
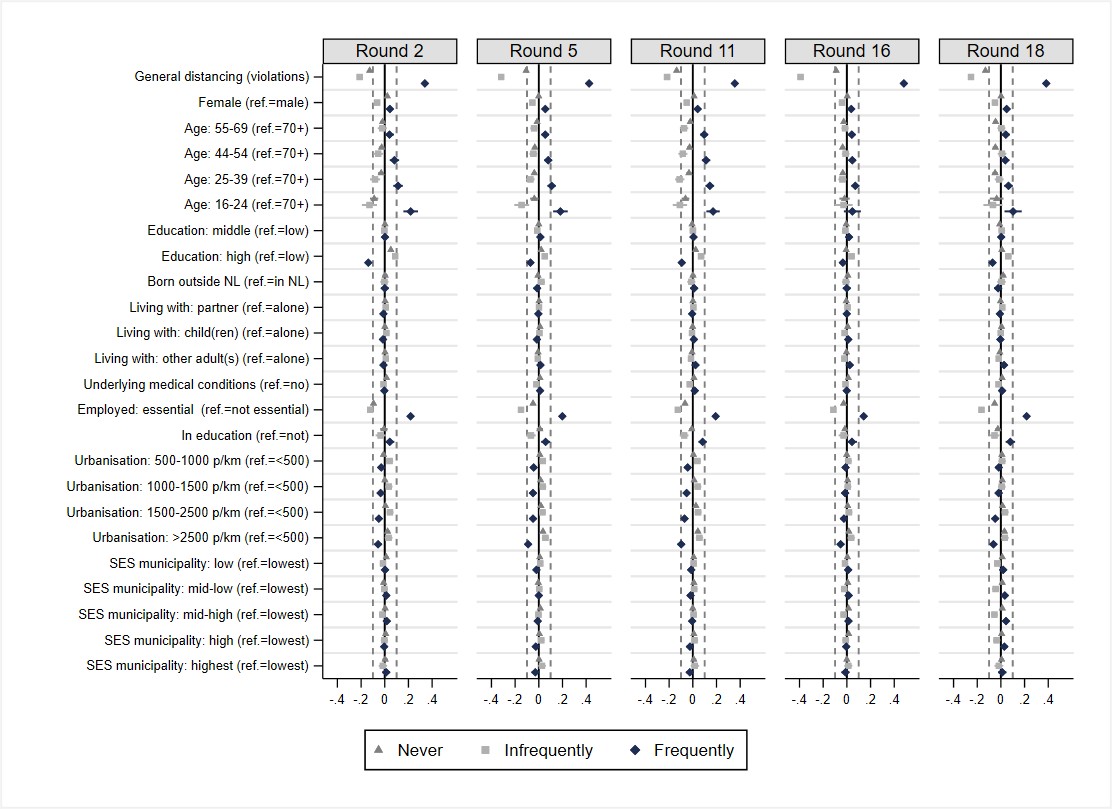
**

**Figure D2a: Associations between sociodemographic factors and physical distancing at work (average marginal effects)**

**
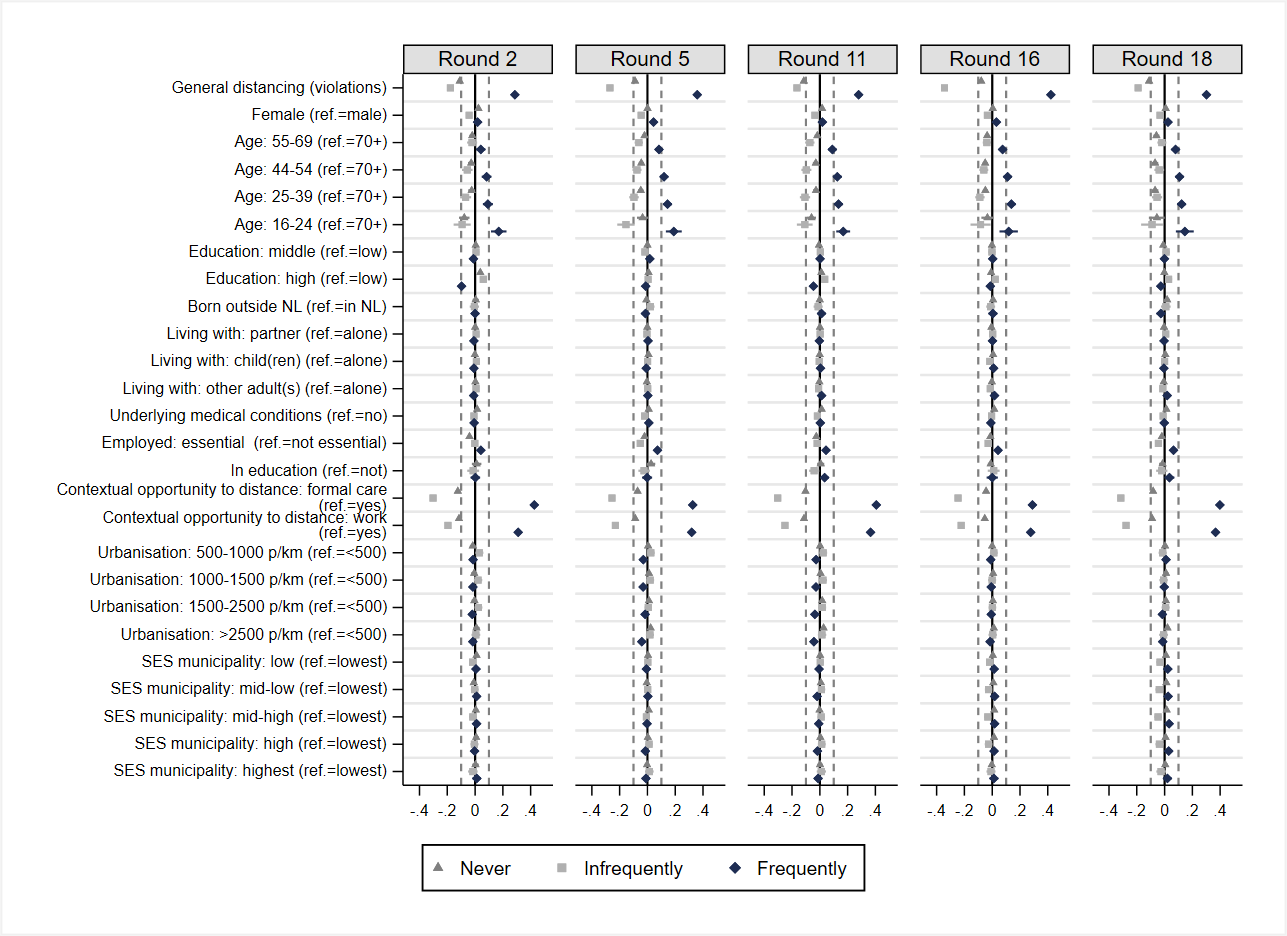
**

**Figure D2b: Associations between sociodemographic factors and physical distancing at work, controlled for work environment (average marginal effects)**

**
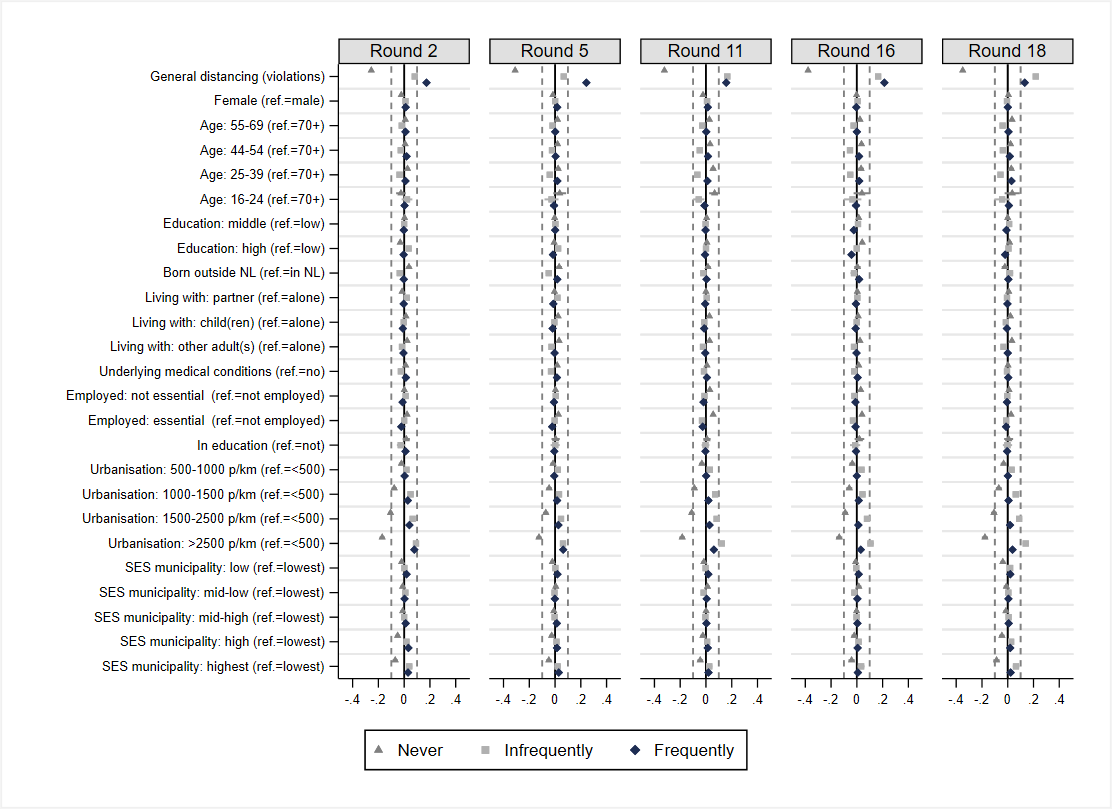
**

**Figure D3: Associations between sociodemographic factors and physical distancing outdoors (average marginal effects)**

**Appendix E: Additional regression models**


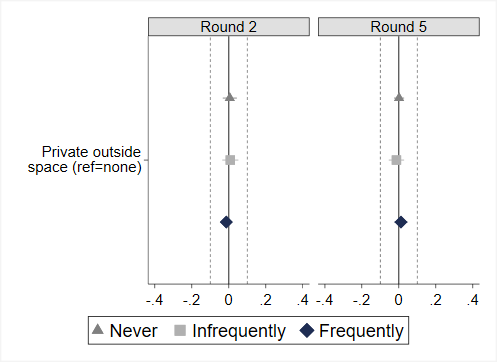


**Figure E1: Associations between having a private outdoor space and physical distancing when friends or family visit at own home (average marginal effects)**

**
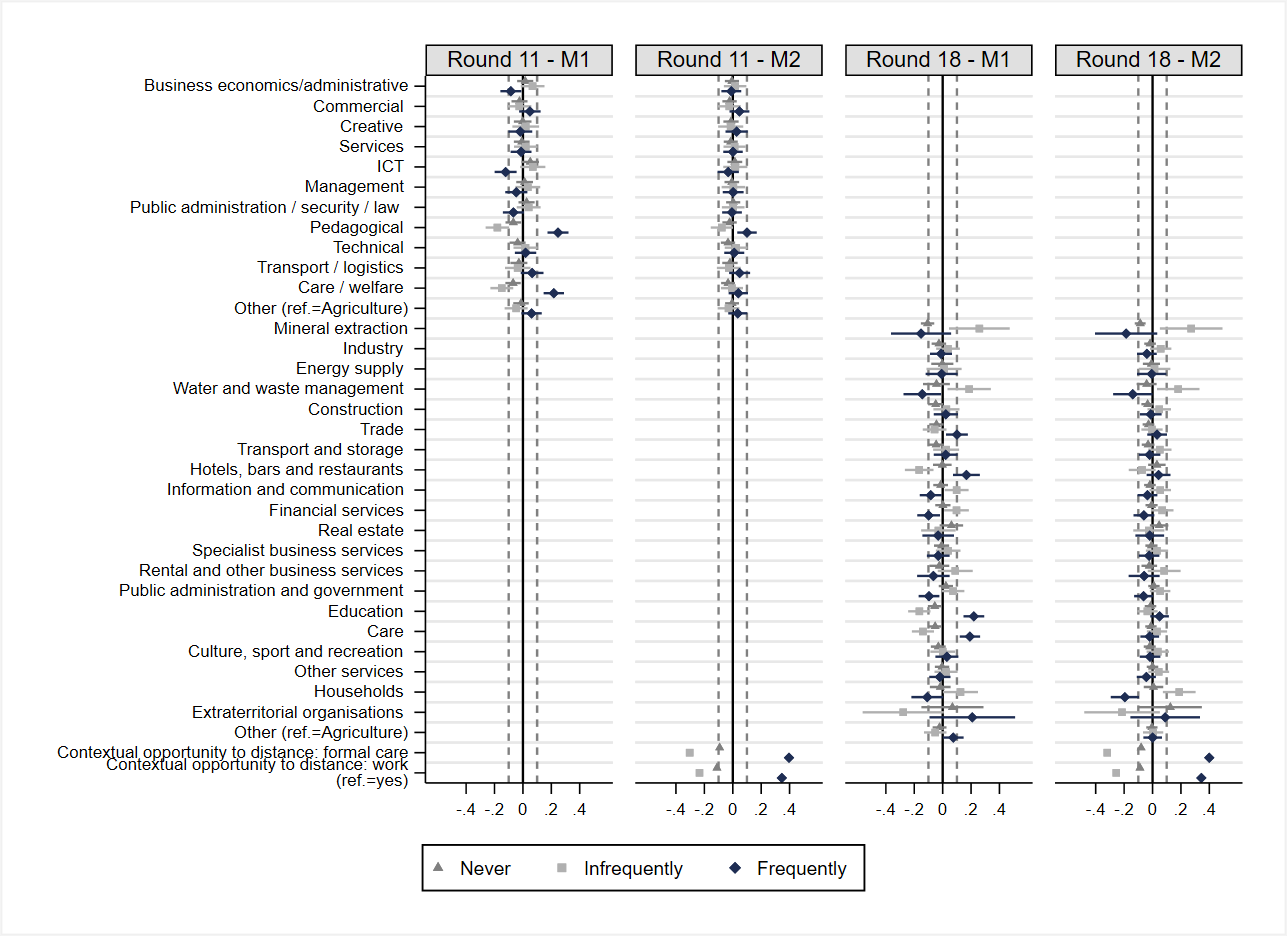
**

**Figure E2: Associations between employment sector and physical distancing at work (average marginal effects)**

1. In each round, the LCA of 2-20 classes resulted in a lower AIC with each additional class. Since these improvements are marginal and models with such large numbers of classes are not interpretable, we stopped estimating additional classes at 20. [↑](#footnote-ref-1)
